# Supplementary material for: Structural basis for the recognition of sulfur in phosphorothioated DNA
Source: Nat Commun. 2018 Nov 8;9:4689. doi: 10.1038/s41467-018-07093-1 (PMC6224610; doi:10.1038/s41467-018-07093-1)
Supplement: Supplementary file 1 — Supplementary Information [file 41467_2018_7093_MOESM1_ESM.pdf]

Structural basis for the recognition of sulfur in phosphorothioated DNA

Liu *et al.*

Supplementary Information

Supplementary Discussion

Why a sulfur atom behaves differently than an oxygen atom and how their difference can lead to specific recognition of the sulfur in PT-DNA

Sulfur and oxygen belong to the same group (group VIA) in the periodic table. The outmost shell electron configuration of oxygen is $2s^2 2p^4$, and the outmost shell electron configuration of sulfur is $3s^2 3p^4$. They both tend to attract two electrons to make their outmost shell electron configuration become $ns^2 np^6$ ($2s^2 2p^6$ for oxygen and $3s^2 3p^6$ for sulfur, respectively). When oxygen forms a covalent bond with phosphorus in normal DNA, it would attract one electron from the phosphorus atom and one electron from the counter-cation (usually Na^+ in physiological condition) to become negatively charged. Sulfur does the same thing. When sulfur forms a covalent bond with phosphorus in PT-DNA, it would attract one electron from the phosphorus atom and one electron from the counter-cation. However, sulfur is much bigger than oxygen. The atomic radius of oxygen is estimated to be 48 picometer while the atomic radius of sulfur is estimated to be 88 picometer. Hence the electronegativity of sulfur is much less than that of oxygen, being 2.58 compared to 3.44 for that of oxygen by the Pauling scale[37]. Therefore, sulfur in PT-DNA carries much less negative charge than oxygen in normal DNA, although it still does carry negative charge and thus can form a salt bridge with the positively charged R117 of ScoMcrA.

Hydrophobicity/hydrophilicity (hydro- means water) is determined by whether an atom (or a molecule) can form hydrogen bond with water or not. Oxygen is highly

electronegative, and thus it carries enough negative charge to form hydrogen bonds with water, and behaves as hydrophilic. In contrast, sulfur is not that electronegative, and it does not possess enough negative charge to form hydrogen bonds with water. Therefore, it behaves as hydrophobic. For example, serine is hydrophilic whereas cysteine (with one oxygen-to-sulfur replacement compared with serine) is much more hydrophobic than serine, and methionine (which contains a sulfur atom) is certainly hydrophobic.

In summary, compared with oxygen in normal DNA, the sulfur atom in PT-DNA is much more hydrophobic. At the same time, it is negatively charged, although the negative charge it carries is much less than that of oxygen. Therefore, it is no surprise that ScoMcrA-SBD employs a hydrophobic pocket to selectively recognize the sulfur atom in PT-DNA, but not the oxygen atom in normal DNA. At the same time, the sulfur atom in PT-DNA is still negatively charged and can form salt bridge with strongly positively charged residues such as R117.

Cleavage by the ScoMcrA HNH domain occurs at a distance of ~23 bp away from the phosphorothioate linkage at the 5′ side of G_{PS}GCC

In a previous work, we detected multiple cleavages of variable efficiency (rectangle in dashed line, **Supplementary Fig. 23a**) flanking the symmetric G_{PS}GCC site by sequencing the cleaved products of a 118 bp 5′-radiolabeled DNA duplex on which the GGCC sites on both strands are phosphorothioated[14]. These results kept it unknown about the orientation of the cleavage sites with respect to the sequence of PT

site for one given DNA molecule. In this regard, 107 bp hemi−PT-DNA on either of strand as well as the full−PT-DNA were synthesized according to the sequence of an internal region of the 118 bp DNA duplex as the substrates for ScoMcrA cleavage assay. Given the cleavage flexibility of ScoMcrA, we use the cleavage site with the highest cut frequency (panel a, rectangle in dashed line) to depict the location of cleavage as well as the length of the cleaved fragments with respect to the PT link. As shown in **Supplementary Fig. 23b**, the DNA duplex of 107 bp that is hemi-phosphorothioated on the top strand was cleaved into two distinct fragments by ScoMcrA, one being ~21 bp, and the other being ~86 bp (**Supplementary Fig. 23b**, lane 3), demonstrating that the cleavage occurred ~20 bp away from the 5′ side of G_{PS}GCC. Similarly, the hemi−PT-DNA on the bottom strand yielded two fragments of ~68 bp and ~39 bp (**Supplementary Fig. 23b**, lane 4), indicative of cleavage at a distance of ~25 bp away from the PT linkage at the 5′ side. By comparison, the fully phosphorothioated DNA on both strands gave a weak band of ~47 bp in addition to the four fragments generated for the hemi−PT-DNA. The length of this new band is exactly in accordance with that of the fragment with dual cuts at flanking sides of G_{PS}GCC/G_{PS}GCC. Cleavage of the hemi−PT-DNA in this result demonstrated that by employing its SBD domain to bind to the G_{PS}GCC site, ScoMcrA orients its HNH domain at the 5′ side of the PT site. The distance between the cleavage sites and PT link for the top and bottom hemi-PT DNA is different, we hypothesize it is possibly due to the DNA sequence specificity of HNH motif of ScoMcrA. DNA sequence specificity for HNH motifs has been reported in I-HumI[1], PacI[2], as well as some HNH

Endonucleases from phage or prophages without cognate recognition domain[3]. The much lower intensity of the 47 bp fragment indicates that most of the DNA molecules are cut only once at either side of PT modification site. The vast majority of DNA molecules is completely uncut, so the chances of getting dual cut molecules is quite low, since the percentage of molecules cut even once is already quite small.

A model of full-length ScoMcrA dimer recognition and cleavage of PT-DNA

If we compare the structure of one protomer of full-length ScoMcrA and that of the SBD-SRA fragment in complex with PT-DNA, they can be superimposed onto each other very well, with a root-mean-square-deviation (RMSD) value of 0.453 Å for 263 atoms being compared (**Supplementary Fig. 24a**). However, if we compare the structures of dimeric full-length ScoMcrA (**Supplementary Fig. 24b**) and dimeric SBD-SRA fragment in complex with PT-DNA (**Supplementary Fig. 24c**), we find that their dimeric arrangements are different. A conformational change is needed for ScoMcrA to rearrange its dimeric assembly upon interaction with PT-DNA (**Supplementary Fig. 24d**).

Using the structure of full-length ScoMcrA and that of the SBD-SRA fragment in complex with PT-DNA, we constructed a model of full-length ScoMcrA dimer in complex with PT-DNA (**Supplementary Fig. 25**). In this model, the residues of the SBD domain from one protomer responsible for phosphorothioate binding such as H116, R117, and P165 (magenta sticks in **Supplementary Fig. 25**), the active site residues H508, N522, and H531 of the HNH domain from the same ScoMcrA

protomer (orange sticks in **Supplementary Fig. 25**), and the active site residues H508′, N522′, and H531′ of the HNH′ domain from the other ScoMcrA protomer (red sticks in **Supplementary Fig. 25**) are all accessible, and they can interact with PT-DNA at the same time in this dimeric configuration. The distance between the SBD-recognition site and the HNH′-contact site on PT-DNA is about 20 base pairs. Therefore, we propose that the full-length ScoMcrA dimer employs the SBD domain of one ScoMcrA protomer to recognize the sulfur atom as well as the G_{PS}GCC core motif, and uses the HNH and HNH′ domains of both ScoMcrA protomers to cleave the two strands of PT-DNA ~20 base pairs away from the SBD-recognition site. This model is fully consistent with our in vitro cleavage assay result (**Supplementary Fig. 23**), and implies that the dimeric organization of ScoMcrA is essential for its biological activity.

If we model two ScoMcrA–SBD domains on the same DNA, their SRGRR loops would clash with each other (**Supplementary Fig. 26a**). In addition, when we measured the binding affinity (K_d) of ScoMcrA–SBD for singly and doubly phosphorothioated DNA, we found that these two K_d values are very similar (**Supplementary Fig. 26b**). Therefore, most likely two ScoMcrA molecules would not bind to two strands of one DNA at the same time. However, the SRGRR loop of ScoMcrA–SBD is quite divergent in SBD homologues from other bacteria, thus we cannot exclude the scenario that other SBD homologues can have two molecules bound to the same DNA at the same time.

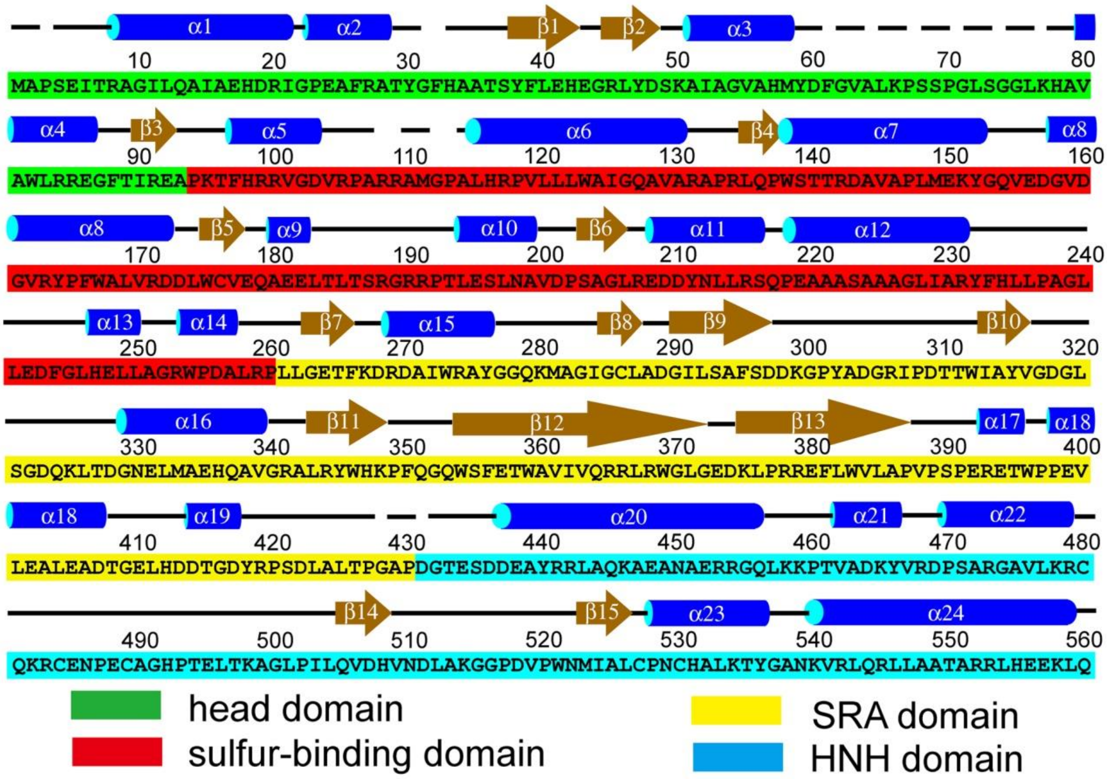

Supplementary Figure 1.

Secondary structure organization of ScoMcrA. The head, SBD, SRA, and HNH domains of ScoMcrA are shaded in green, red, yellow, and cyan, respectively. Residues with poor electron density and not included in the final model are indicated by dashed lines.

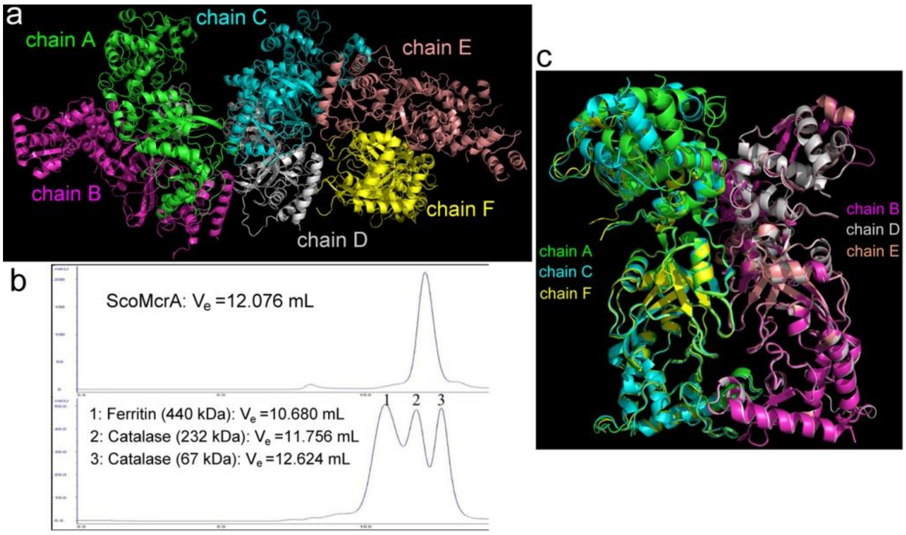

Supplementary Figure 2.

Three ScoMcrA dimers are present in the asymmetric unit of the structure of full-length (FL) ScoMcrA alone. (**a**) There are six molecules of ScoMcrA in the asymmetric unit of the FL ScoMcrA structure, colored in green, magenta, cyan, grey, wheat, and yellow for chains A, B, C, D, E, and F, respectively. They are assembled into three dimers: A-B pair, C-D pair, and E-F pair. (**b**) ScoMcrA forms a dimer in solution. The elution profile of FL ScoMcrA from Superdex 200 gel filtration chromatography is shown. The elution volumes of standard protein molecular weight markers are also indicated. Theoretical molecular weights for a monomer and a dimer of ScoMcrA are 62.08 kDa and 124.15 kDa, respectively. (**c**) Comparison of the three dimers of ScoMcrA (A-B pair, C-D pair, and E-F pair) in the asymmetric unit.

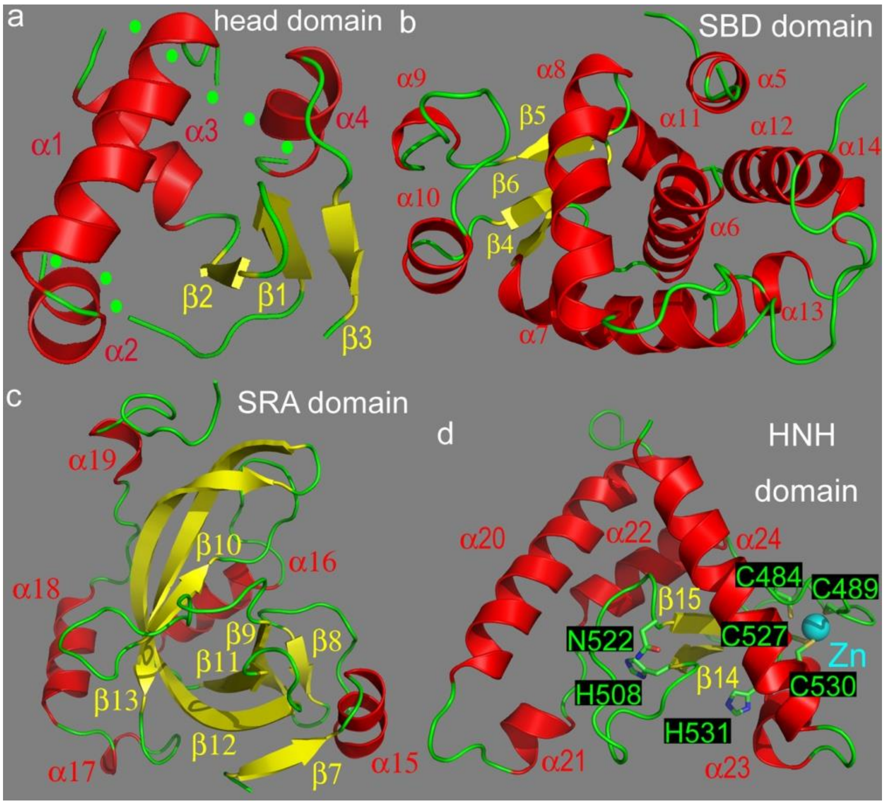

Supplementary Figure 3.

Structures of the four individual domains of ScoMcrA. (**a**) Structure of the head domain of ScoMcrA. α helices, β strands, and loops are colored in red, yellow, and green, respectively. Secondary structure elements are labeled. Residues with poor electron density and thus not included in the final model are indicated with green dotted lines. (**b**) Structure of the SBD domain of ScoMcrA. (**c**) Structure of the SRA domain of ScoMcrA. (**d**) Structure of the HNH domain of ScoMcrA. Side chains of the active site residues H508, N522, and H531 critical for the endonuclease activity of ScoMcrA and the zinc-chelating residues C484, C489, C527, and C530 are shown. Carbon, nitrogen, oxygen, and sulfur atoms are colored in green, blue, red, and gold, respectively. The zinc ion associated with the HNH domain is shown as a cyan sphere.

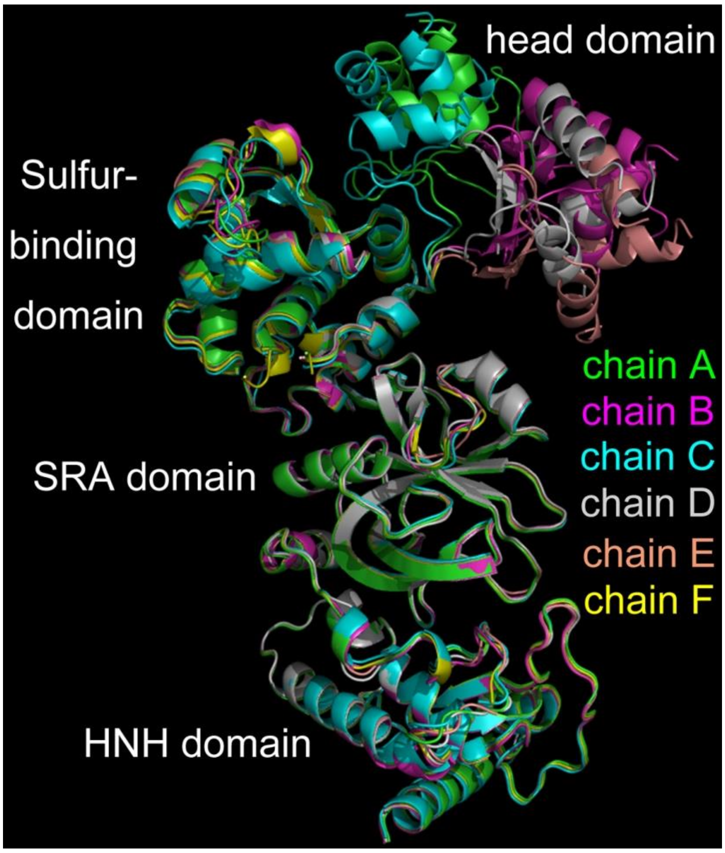

Supplementary Figure 4.

The head domain is highly flexible with respect to the rest of the ScoMcrA molecule. The six molecules of ScoMcrA in the asymmetric unit of the FL ScoMcrA structure are superimposed. The SBD domains, SRA domains, and HNH domains of the six molecules can be superimposed relatively well, whereas the positions of the head domains from different ScoMcrA molecules exhibit considerable variations. Note that there is also some limited flexibility between the SBD domain and the SRA domain, as well as between the SRA domain and the HNH domain.

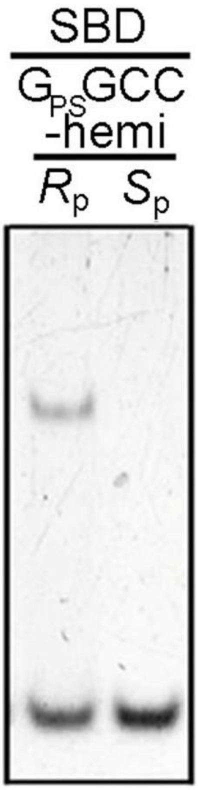

Supplementary Figure 5.

The SBD domain of ScoMcrA interacts specifically with the *R*p, but not the *S*p, form of hemi–phosphorothioated DNA.

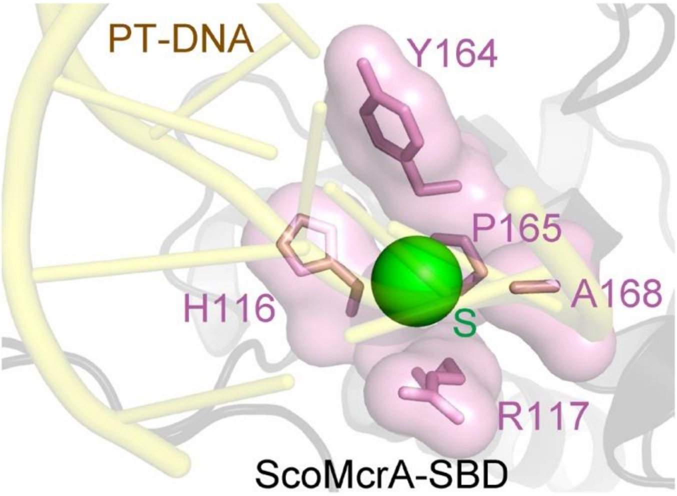

Supplementary Figure 6.

View of the sulfur-binding cavity of ScoMcrA–SBD from the top. Sulfur-recognizing residues of ScoMcrA–SBD are shown in stick as well in surface representations.

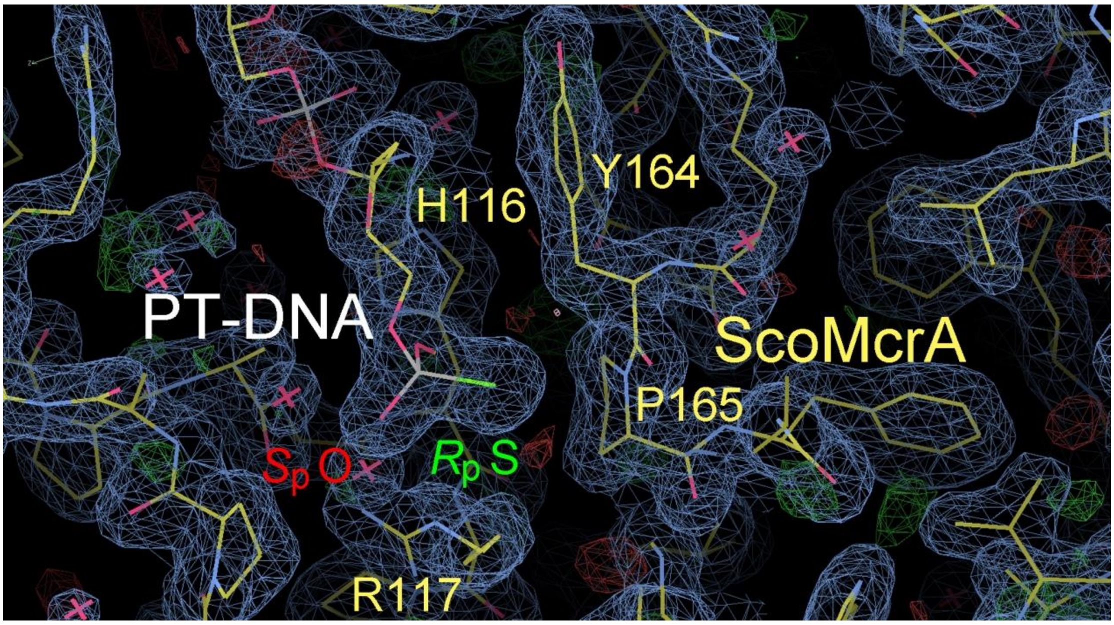

Supplementary Figure 7.

A portion of the electron density map of ScoMcrA−SBD in complex with PT-DNA around the *R*p sulfur atom and the *S*p oxygen atom. The $2F_o{-}F_c$ electron density map around the *R*p sulfur atom and the *S*p oxygen atom of PT-DNA is shown at the 1.0 σ contour level (cyan). The ScoMcrA−SBD and PT-DNA structure models are superimposed onto the electron density map. In the structure models, sulfur, carbon, oxygen, and nitrogen atoms are colored in green, yellow, red, and blue, respectively. Water molecules are denoted as red crosses. The $F_o{-}F_c$ difference electron density map is also shown at the 2.5 σ contour level, with green and red representing positive and negative difference densities, respectively.

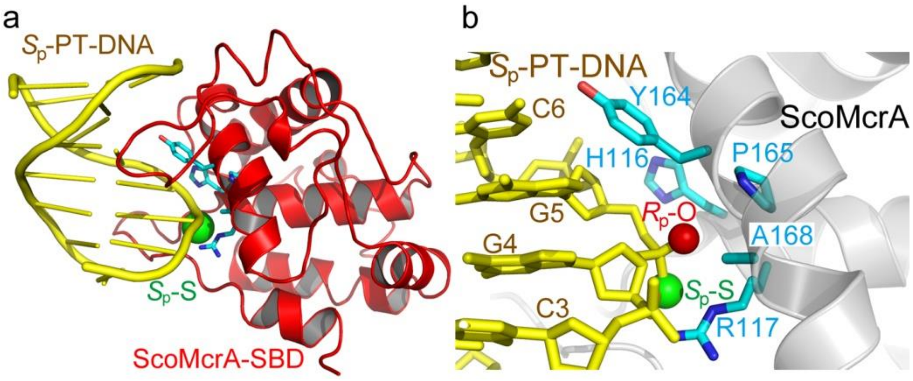

Supplementary Figure 8.

In PT-DNA with the sulfur atom in the S_p configuration, the S_p sulfur atom would be in an unsuitable position that awkwardly interacts with ScoMcrA.

(**a**) ScoMcrA–SBD with S_p PT-DNA docked. The sulfur atom in the S_p configuration does not fit into the sulfur-binding cavity of ScoMcrA–SBD. (**b**) Close-up view of the putative interface between ScoMcrA–SBD and S_p PT-DNA. The hydrophilic oxygen atom, rather than the relatively hydrophobic sulfur atom, in the R_p configuration is located in the non-polar surface cavity of ScoMcrA–SBD lined with the pyrolidine ring of P165, the β-methylene groups of Y164 and H116, the β-methyl group of A168, which is unfavorable for the interaction.

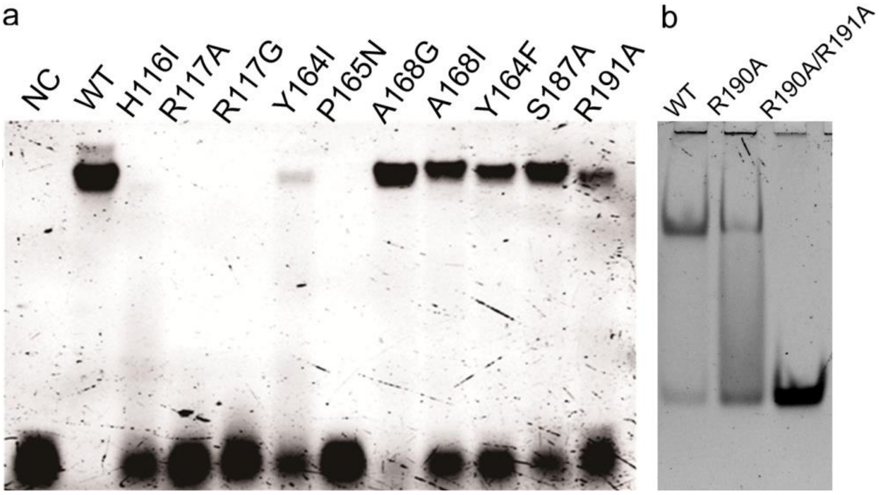

Supplementary Figure 9.

Mutations of key sulfur-recognizing residues or base-contacting residues disrupted or decreased the association between ScoMcrA–SBD and PT-DNA as analyzed by the EMSA assay. (**a**) Point mutants of H116I, R117A, R117G, Y164I, P165N, A168G, A168I, Y164F, S187A, and R191A were analyzed by the EMSA assay along with the wild-type ScoMcrA−SBD protein. NC, control without any protein added. (**b**) Single point mutant R190A and double point mutant R190A/R191A were analyzed by the EMSA assay along with the wild-type ScoMcrA−SBD protein.

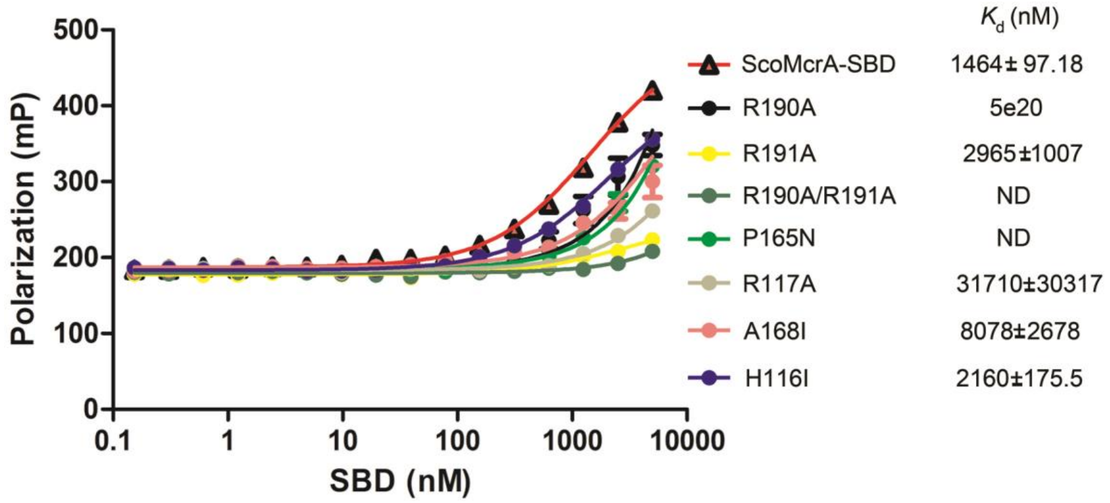

Supplementary Figure 10.

Fluorescence polarization assay showed that mutations of key sulfur-recognizing residues or base-contacting residues disrupted or decreased the association between ScoMcrA–SBD and PT-DNA. ND, the K_d value was too large to be determined because the measured polarization values were out of range. The error values were the standard error of the mean, and were calculated from two to four parallel experiments.

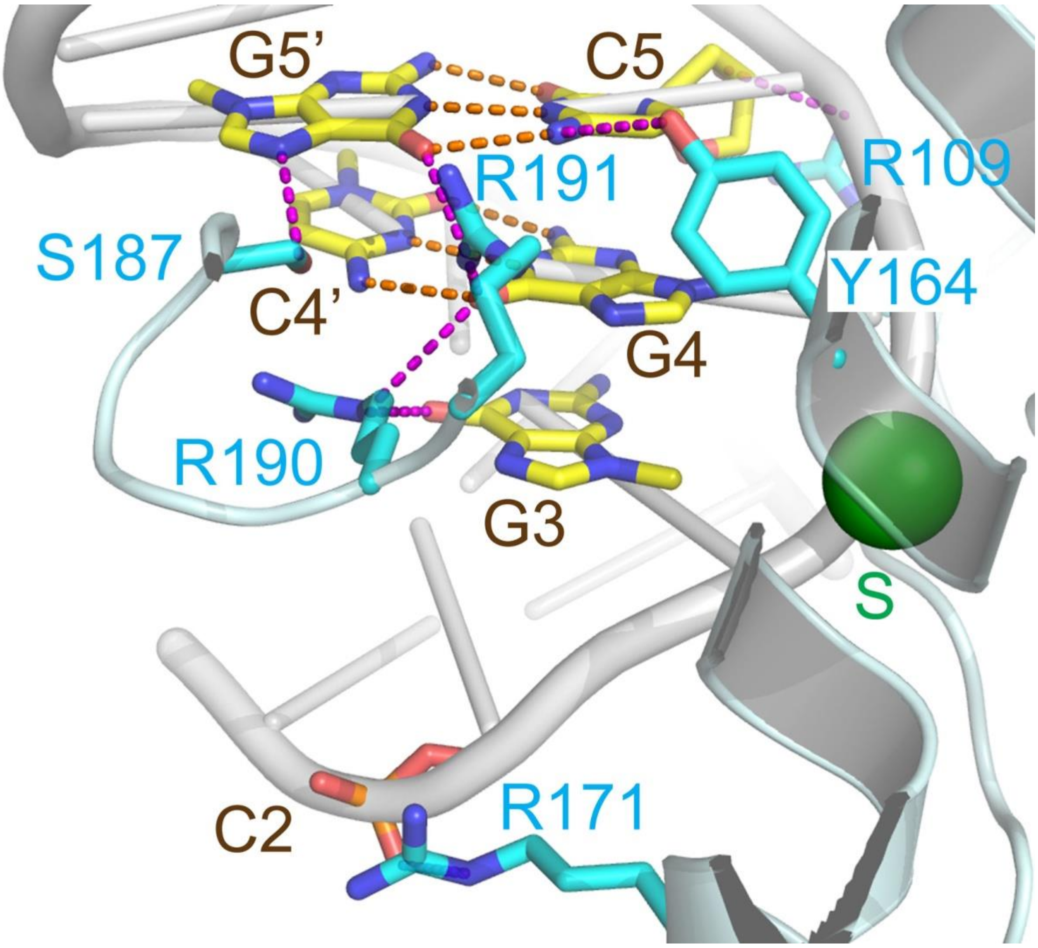

Supplementary Figure 11.

The "S^{187}RGRR^{191} loop" of ScoMcrA inserts into the major groove of PT-DNA and provides base-specific interactions with the G$_{PS}$GCC core sequence. The hydrogen bonds between ScoMcrA and PT-DNA are represented by magenta dashed lines, and those between base pairs of PT-DNA are shown as orange dashed lines.

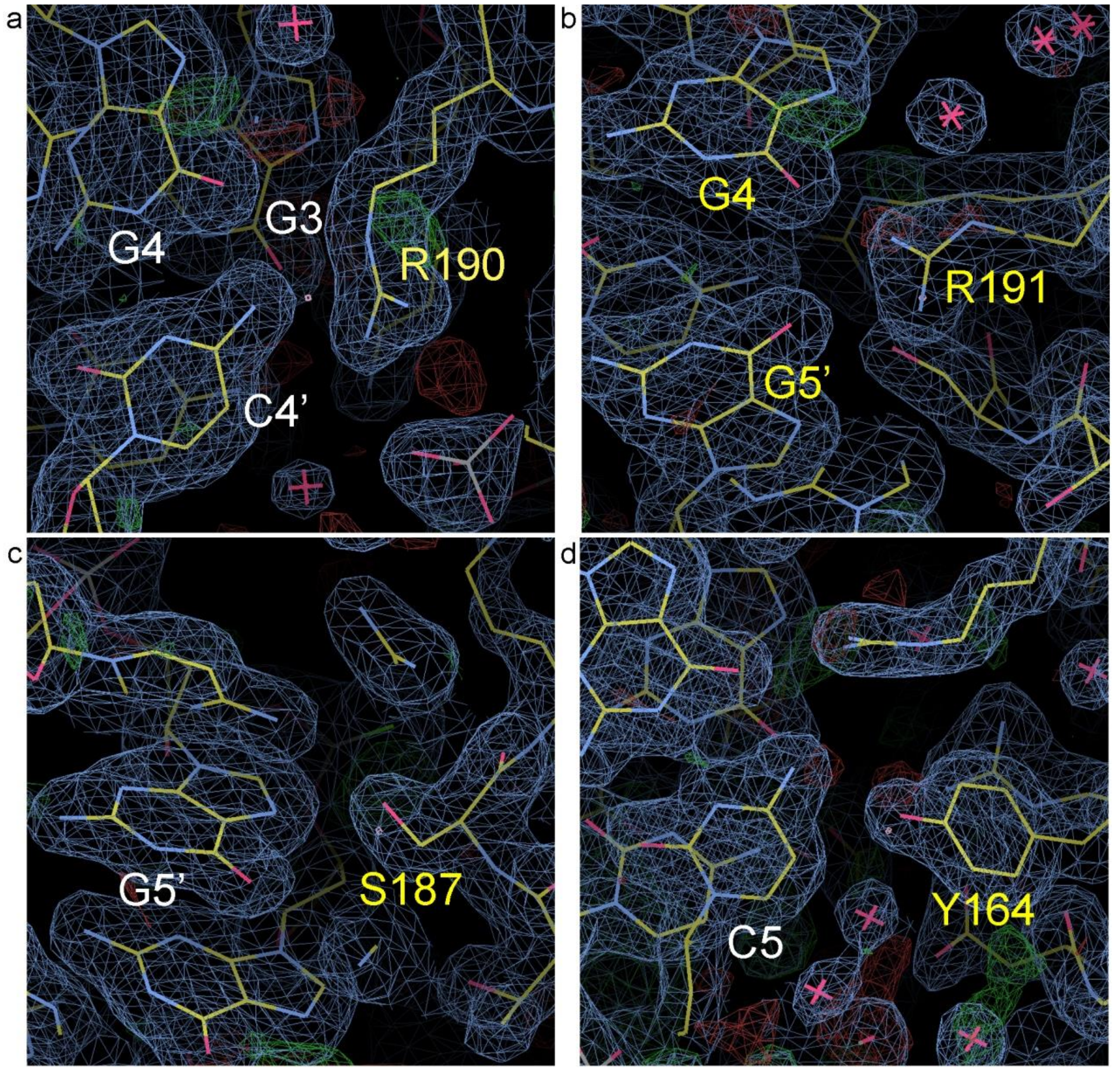

Supplementary Figure 12.

Portions of the electron density map of ScoMcrA−SBD in complex with PT-DNA around the "S[187]RGRR[191] loop" of ScoMcrA−SBD. The $2F_o-F_c$ electron density maps around ScoMcrA-R190 (**a**), R191 (**b**), S187 (**c**), and Y164 (**d**) are shown at the 1.0 σ contour level (cyan). The ScoMcrA−SBD and PT-DNA structure models are superimposed onto the electron density map. In the structure models, carbon, oxygen, and nitrogen atoms are colored in yellow, red, and blue, respectively. Water molecules are denoted as red crosses. The F_o-F_c difference electron density map is also shown at the 2.5 σ contour level, with green and red representing positive and negative difference densities, respectively.

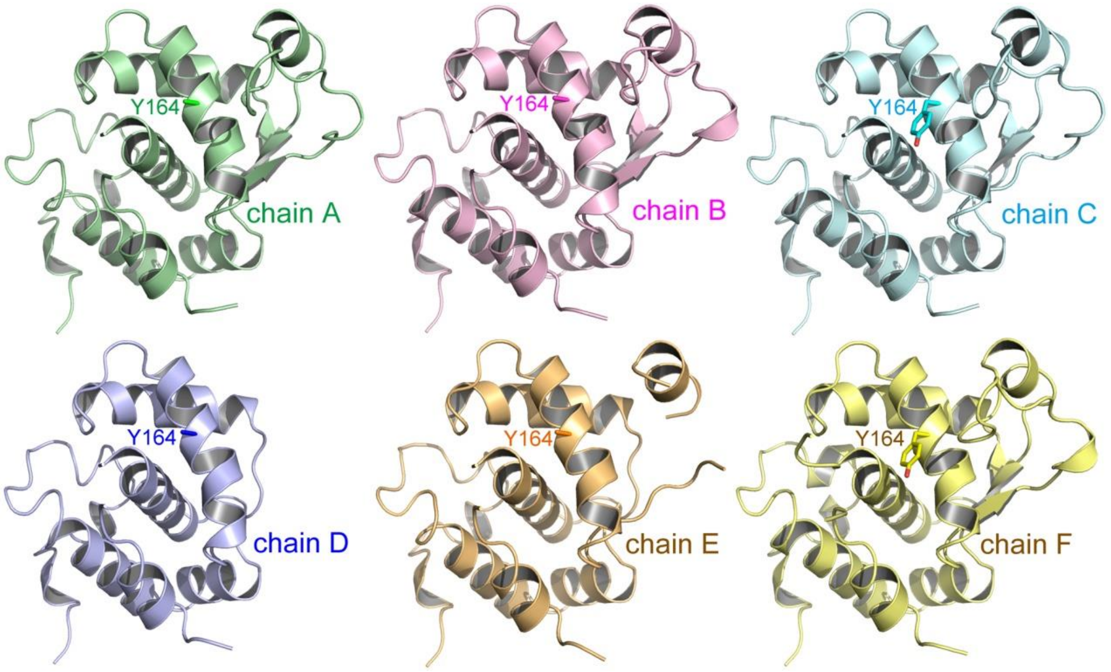

Supplementary Figure 13.

Comparison of the conformations of Y164 in the six different molecules of ScoMcrA in the asymmetric unit of the crystal structure of full-length ScoMcrA by itself. Only in chain C and chain F was the electron density of Y164 clear enough to unambiguously observe the hydroxyphenyl groups of Y164, which are both in the “closed” conformation. The hydrophobic phenyl ring of the side-chain of Y164 covers the opening of the non-polar sulfur-binding cavity. In chains A, B, D, and E, the electron densities of the side-chain of Y164 were not clear, and Y164 was modeled as alanines in these chains. This observation indicates that Y164 of ScoMcrA is flexible and samples a variety of conformations when ScoMcrA is not in complex with PT-DNA.

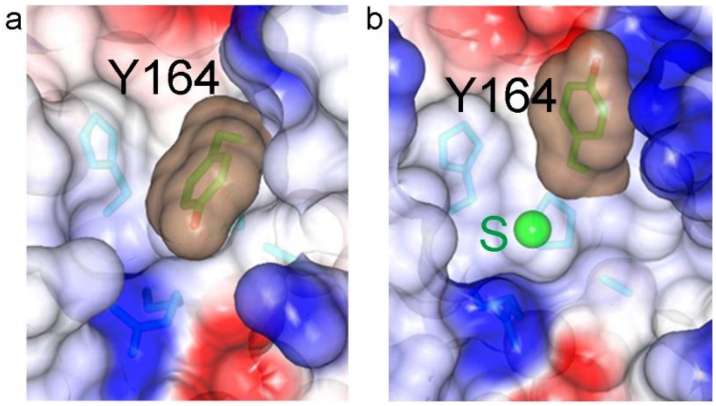

Supplementary Figure 14.

Front views for the “closed” state of Y164 in the PT-DNA−unbound ScoMcrA-SBD and for the “open” state of Y164 in the PT-DNA−bound ScoMcrA-SBD. This suggests that when not in complex with PT-DNA, Y164 of ScoMcrA-SBD is flexible and samples a variety of conformations, including the “closed” state which the hydroxyphenyl ring of Y164 covers the opening of the sulfur-binding pocket. On the other hand, binding to PT-DNA (both to the sulfur atom and to the G_{PS}GCC core sequence) induces the flipping of hydroxyphenyl ring of Y164 and stabilizes it in the “open” state. (**a**) In the PT-DNA−unbound ScoMcrA-SBD, Y164 exists in the “closed” state, and its phenyl ring covers the sulfur-binding cavity of ScoMcrA-SBD. The electrostatic surface of ScoMcrA-SBD is shown. Y164 is displayed both in stick and space-filling representations. (**b**) In the PT-DNA−bound ScoMcrA-SBD, Y164 exists in the “open” state. The hydroxyphenyl group of its side-chain rotates away to allow the sulfur atom of PT-DNA to access the sulfur-binding cavity of ScoMcrA-SBD. The sulfur atom of PT-DNA is shown as a green sphere. The rest of PT-DNA is omitted for clarity.

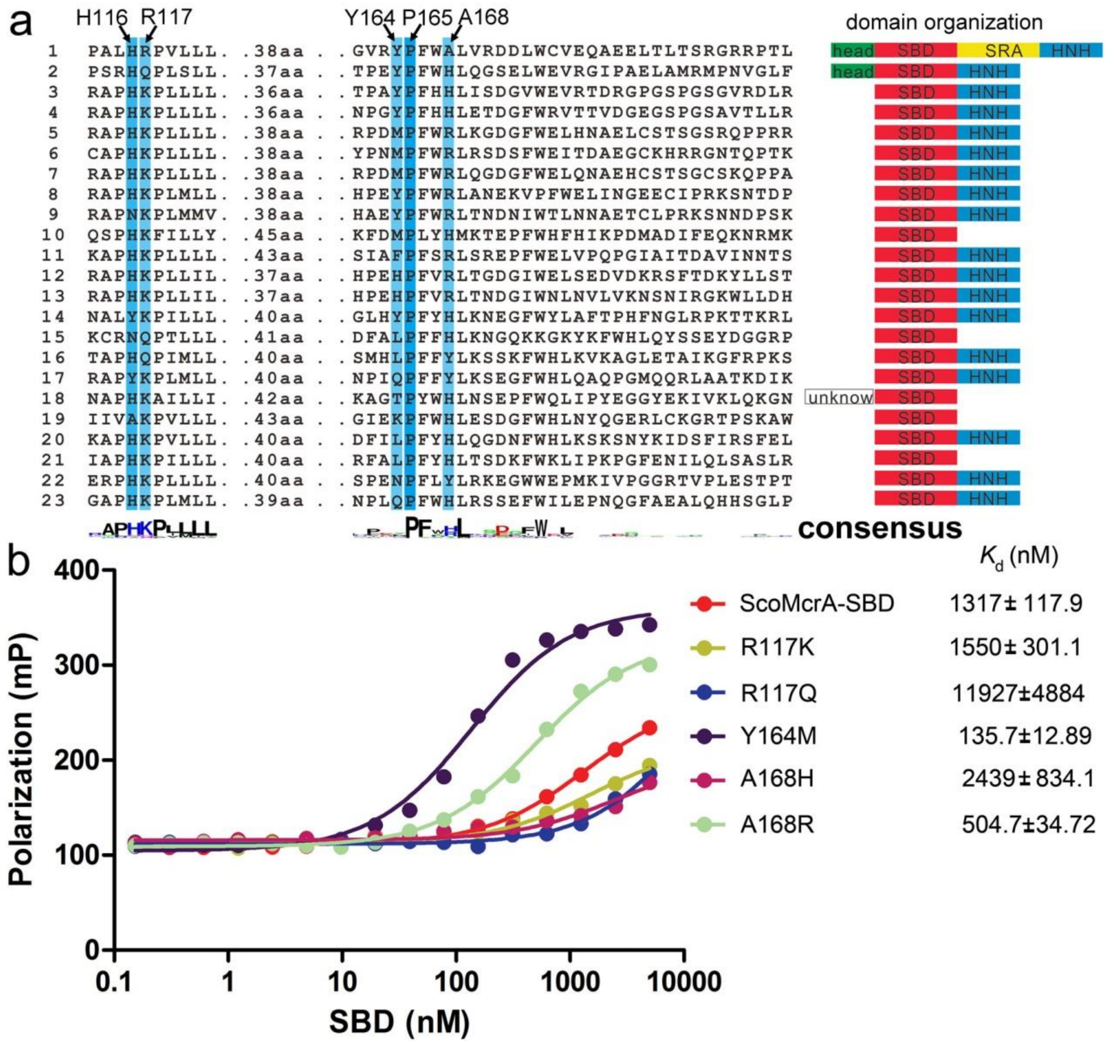

Supplementary Figure 15.

Multiple sequence alignment of representative ScoMcrA–SBD homologues from various bacteria, and measurement of the dissociation constant of various point mutants of ScoMcrA–SBD for PT-DNA. (**a**) Some of the sulfur-recognizing residues are highly conserved. The consensus sequence is shown at the bottom. Domain organizations of these ScoMcrA homologues are shown on the right. The protein accession numbers and the names of the organisms they derive from are listed in **Supplementary Table 3**. (**b**) Mutation of R117 did not appreciably affect, while mutations of R117Q or A168H compromised the binding between ScoMcrA–SBD and PT-DNA. Mutations of Y164M and A168R somehow increased

the binding affinity of ScoMcrA–SBD for PT-DNA. The error values were the standard error of the mean, and were calculated from two to four parallel experiments.

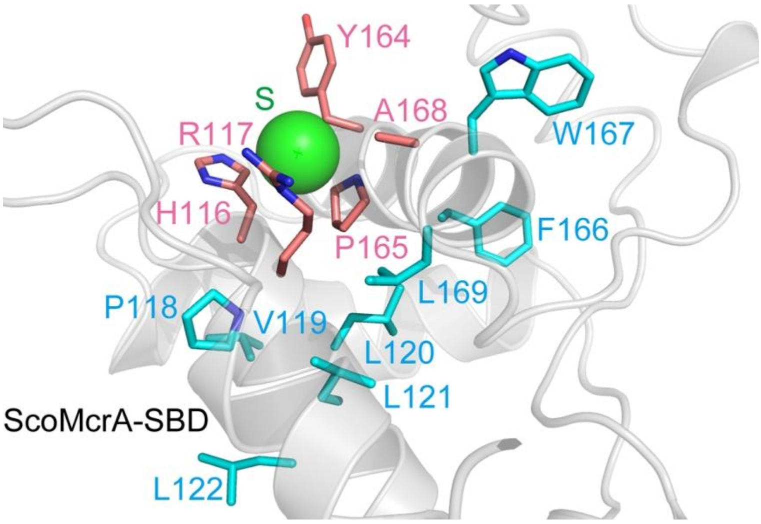

Supplementary Figure 16.

Conserved residues P118, V119, L120, L121, F166, W167, L169, and W175 of ScoMcrA participate in forming the hydrophobic core of the SBD domain of ScoMcrA.

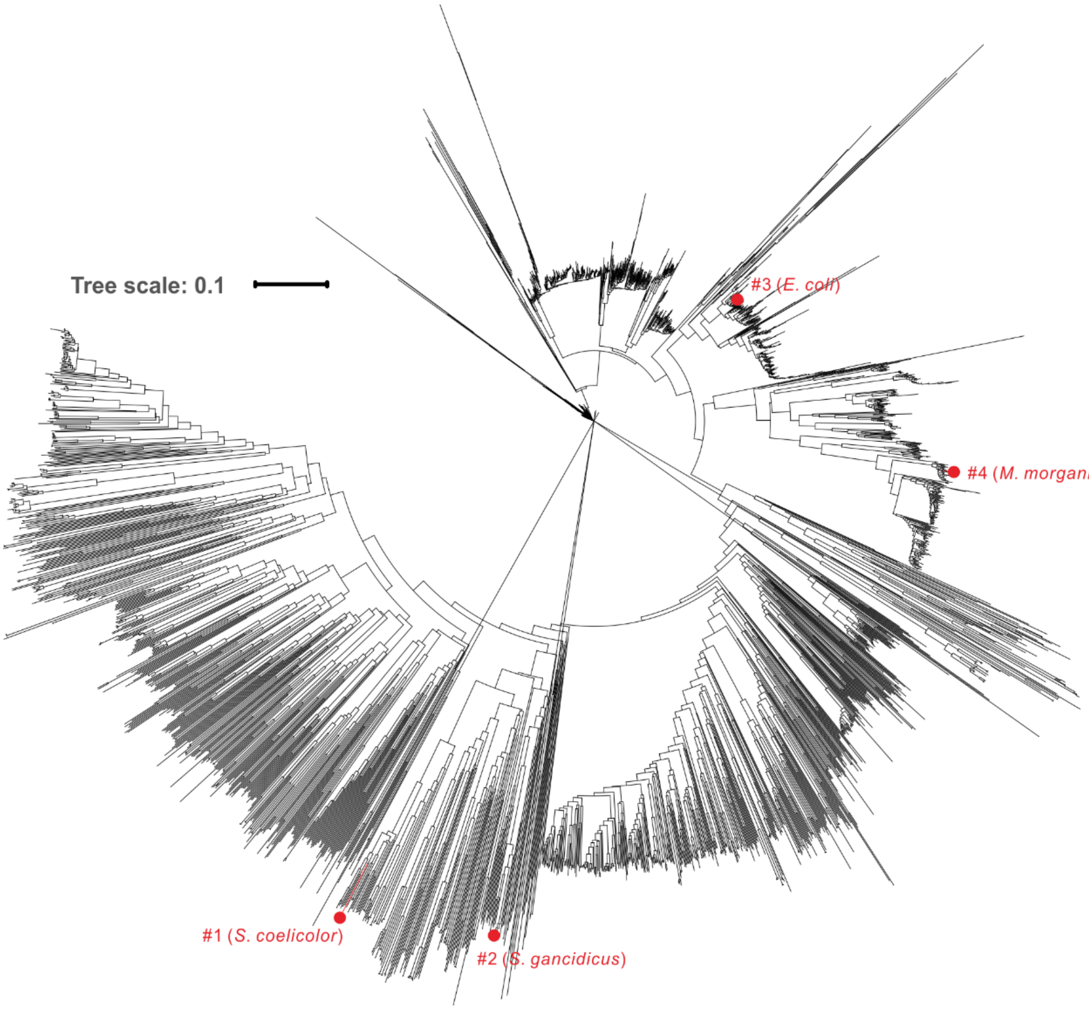

Supplementary Figure 17.

Phylogenic tree for ScoMcrA–SBD homologues. Phylogenic tree for 2,761 ScoMcrA−SBD homologues constructed based on amino acid sequences. Homologues from *Streptomyces coelicolor* (#1), *Streptomyces gancidicus* (#2), *Escherichia coli* (#3), and *Morganella morganii* (#4) are marked with red dots.

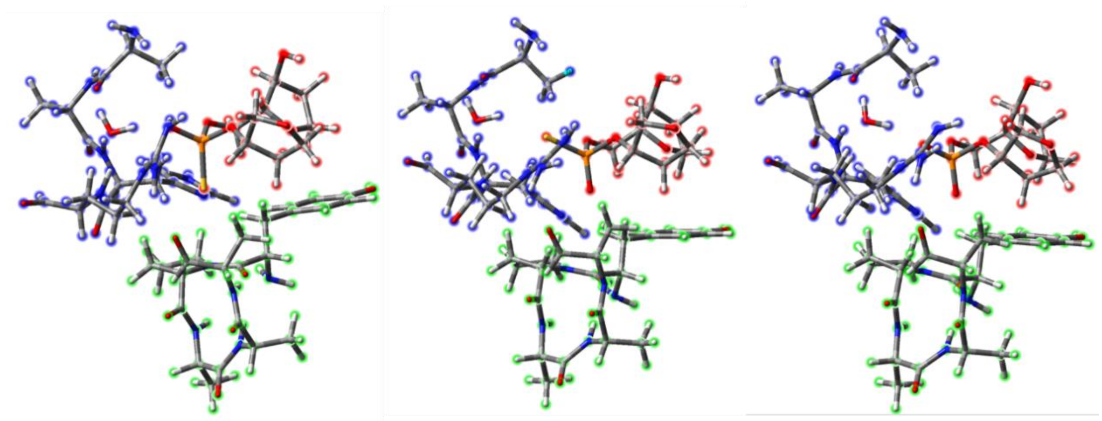

Supplementary Figure 18.

The optimized R_P-, S_P-phosphorothioate and normal phosphate in complex with α-helices 5 and 8 of SBD protein are shown in the left, middle, and right panels, respectively. Phosphorothioate/phosphate, α-helices 5 and 8 were drawn in red, blue, and green, respectively.

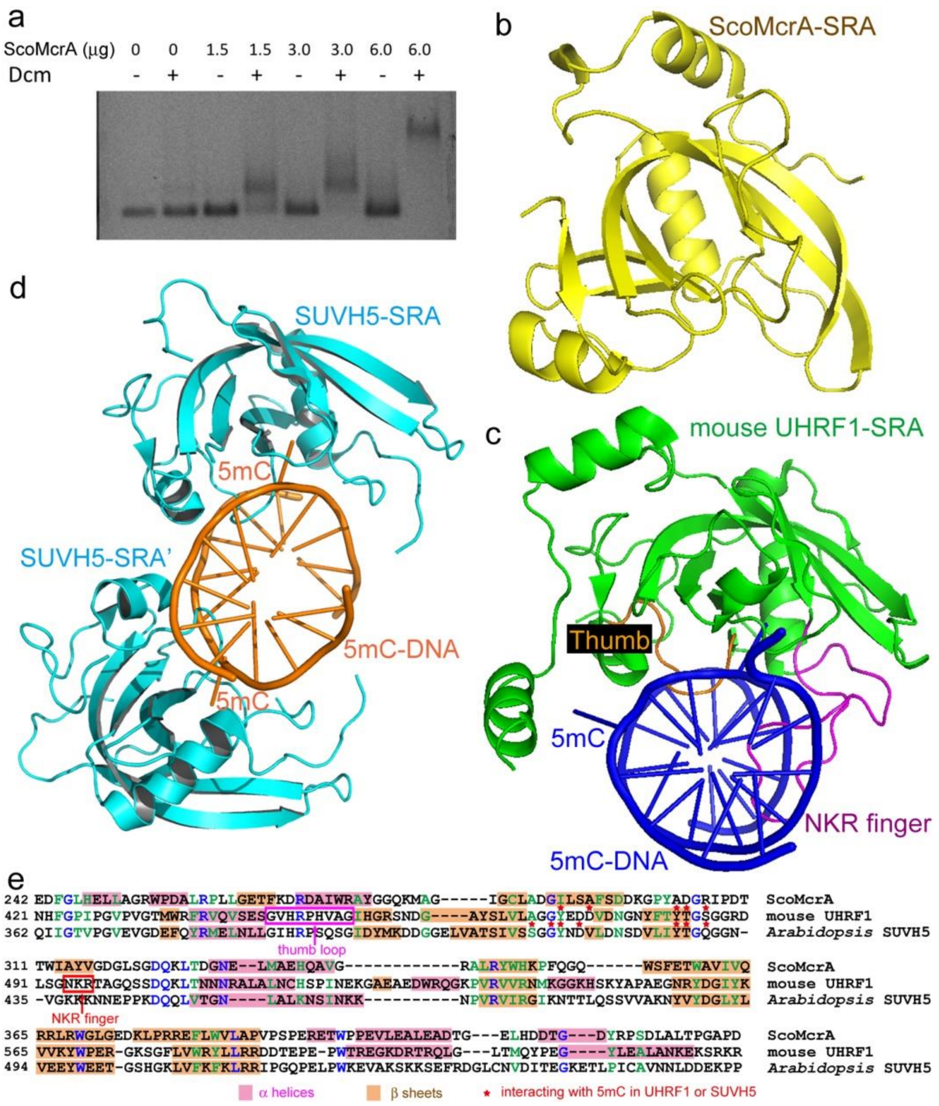

Supplementary Figure 19.

Structural comparison of the SRA domain of ScoMcrA with that of UHRF1 and of SUVH5. (**a**) ScoMcrA interacts with DNA methylated by Dcm, as analyzed by the EMSA assay. (**b**) Structure of the SRA domain of ScoMcrA. (**c**) Structure of the SRA domain of mouse UHRF1 in complex with hemi-methylated DNA (PDB code: 2ZO0)[19]. The "NKR finger" and "thumb loop" motifs of UHRF1 that participate in binding methylated DNA are indicated. Note that the "NKR finger" and "thumb loop" features are absent in the structure of the SRA domain of ScoMcrA. (**d**) Structure of the SRA domain of *Arabidopsis* SUVH5 in complex with fully methylated DNA (PDB code: 3Q0B)[21]. (**e**) Structure-based sequence alignment of the

SRA domains of ScoMcrA, mouse UHRF1, and *Arabidopsis* SUVH5. α helices and β sheets are shaded in pink and salmon, respectively. Identical or similar residues among ScoMcrA, UHRF1, and SUVH5 are colored in blue or green, respectively. The 5-methyl cytosine (5mC)-interacting residues of UHRF1 and SUVH5 are marked by red stars. The "NKR finger" and "thumb loop" motifs of UHRF1 are indicated by red and magenta squares, respectively.

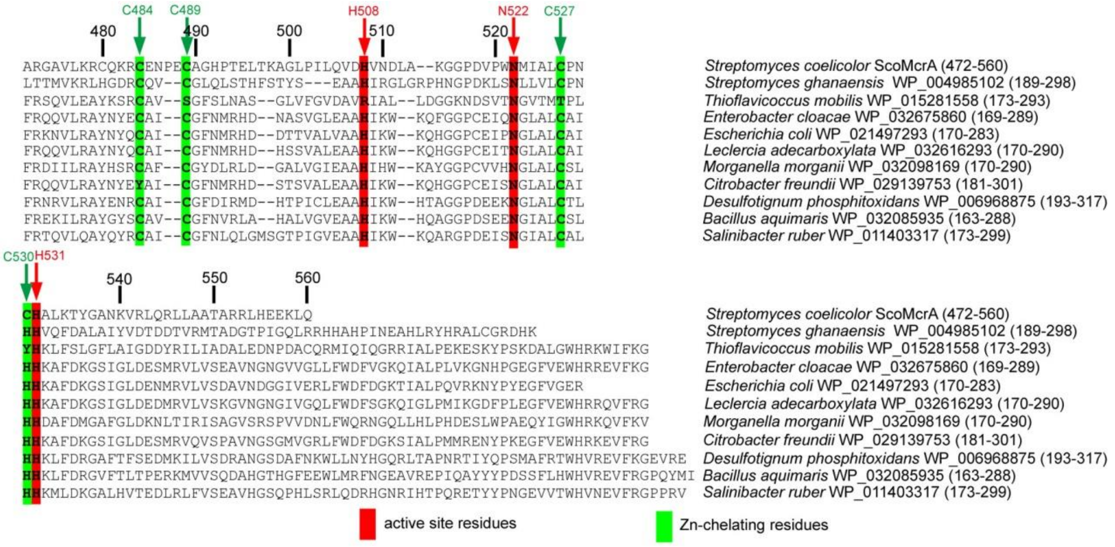

Supplementary Figure 20.

Multiple sequence alignment of the HNH domains of ScoMcrA homologues from various bacteria. Residues corresponding to the active site residues H508, N522, and H531 important for the endonuclease activity of ScoMcrA are highlighted in red, while those corresponding to the four zinc-chelating residues C484, C489, C527, and C530 are highlighted in green.

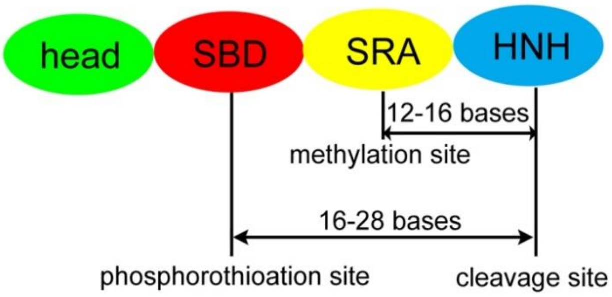

Supplementary Figure 21.

A schematic model explaining that ScoMcrA cleaves DNA farther from the phosphorothioation site than from the methylation site. The spatial positions of the SBD, SRA, and HNH domains of ScoMcrA are linearly organized. The physical distance between the SBD and the HNH domains is larger than that between the SRA and HNH domain. Since the SBD, SRA, and HNH domains are responsible for recognizing PT-DNA, associating with methylated DNA, and performing double strand cleavage on DNA, respectively, this provides an explanation that the distance between the phosphorothioation and ScoMcrA cleavage sites on PT-DNA is larger than that between the methylation and ScoMcrA cleavage sites on methylated DNA[14].

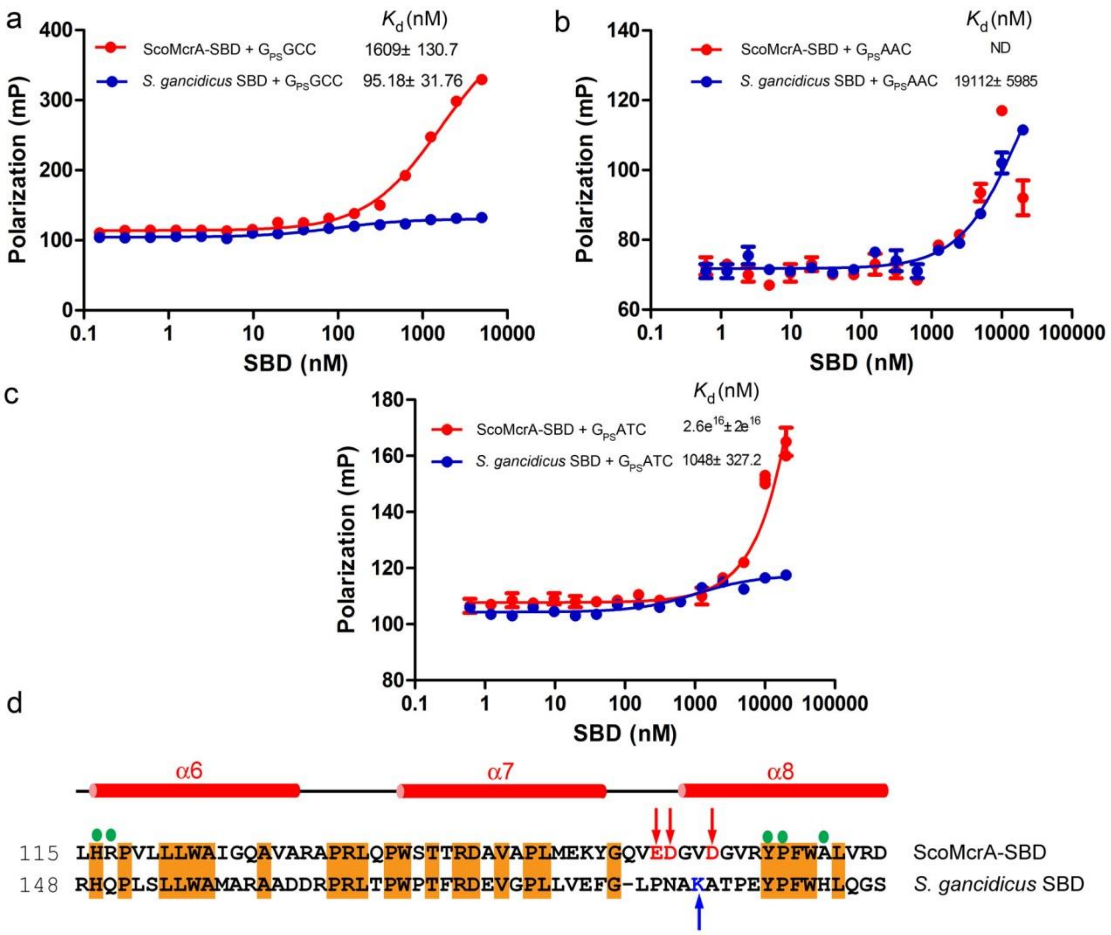

Supplementary Figure 22.

Streptomyces gancidicus **SBD homologue associated with PT-DNA harboring core sequence of GPSGCC, GPSAAC, or GPSATC; whereas ScoMcrA–SBD only interacted with GPSGCC PT-DNA.** (**a**) Both the *S. gancidicus* SBD homologue and ScoMcrA–SBD associated with PT-DNA harboring core sequence of GPSGCC. The error values were the standard error of the mean, and were calculated from two to four parallel experiments. (**b**) The *S. gancidicus* SBD homologue associated with PT-DNA harboring core sequence of GPSAAC, but ScoMcrA–SBD did not. (**c**) The *S. gancidicus* SBD homologue associated with PT-DNA harboring core sequence of GPSATC, but there was almost no interaction between ScoMcrA–SBD and GPSATC PT-DNA. (**d**) Negatively charged residues E156, D157, and D160, present in ScoMcrA–SBD but absent in the *S. gancidicus*

SBD homologue may decrease the interaction between ScoMcrA–SBD and PT-DNA so that ScoMcrA–SBD is unable to associate with $G_{PS}AAC$ and $G_{PS}ATC$; whereas positively charged residue K191 present in the *S. gancidicus* SBD homologue but absent in ScoMcrA–SBD may increase the interaction between the *S. gancidicus* SBD homologue and PT-DNA. Sequence comparison between ScoMcrA–SBD and the *S. gancidicus* SBD homologue is shown. The three negatively charged residues E156, D157, and D160, present in ScoMcrA–SBD but absent in the *S. gancidicus* SBD homologue, are shown in red and marked by red arrows. The positively charged residue K191, present in the *S. gancidicus* SBD homologue but absent in ScoMcrA–SBD, is shown in blue and marked by a blue arrow. The sulfur-binding residues H116, R117, Y164, P165, and A168 in ScoMcrA–SBD are indicated with green ovals. Conserved residues are shaded in orange. Secondary structures and starting residue numbers are indicated above and on the left of the protein sequences, respectively. The "SRGRR loop" of ScoMcrA–SBD exists between α helices α9 and α10, and is not shown in the figure.

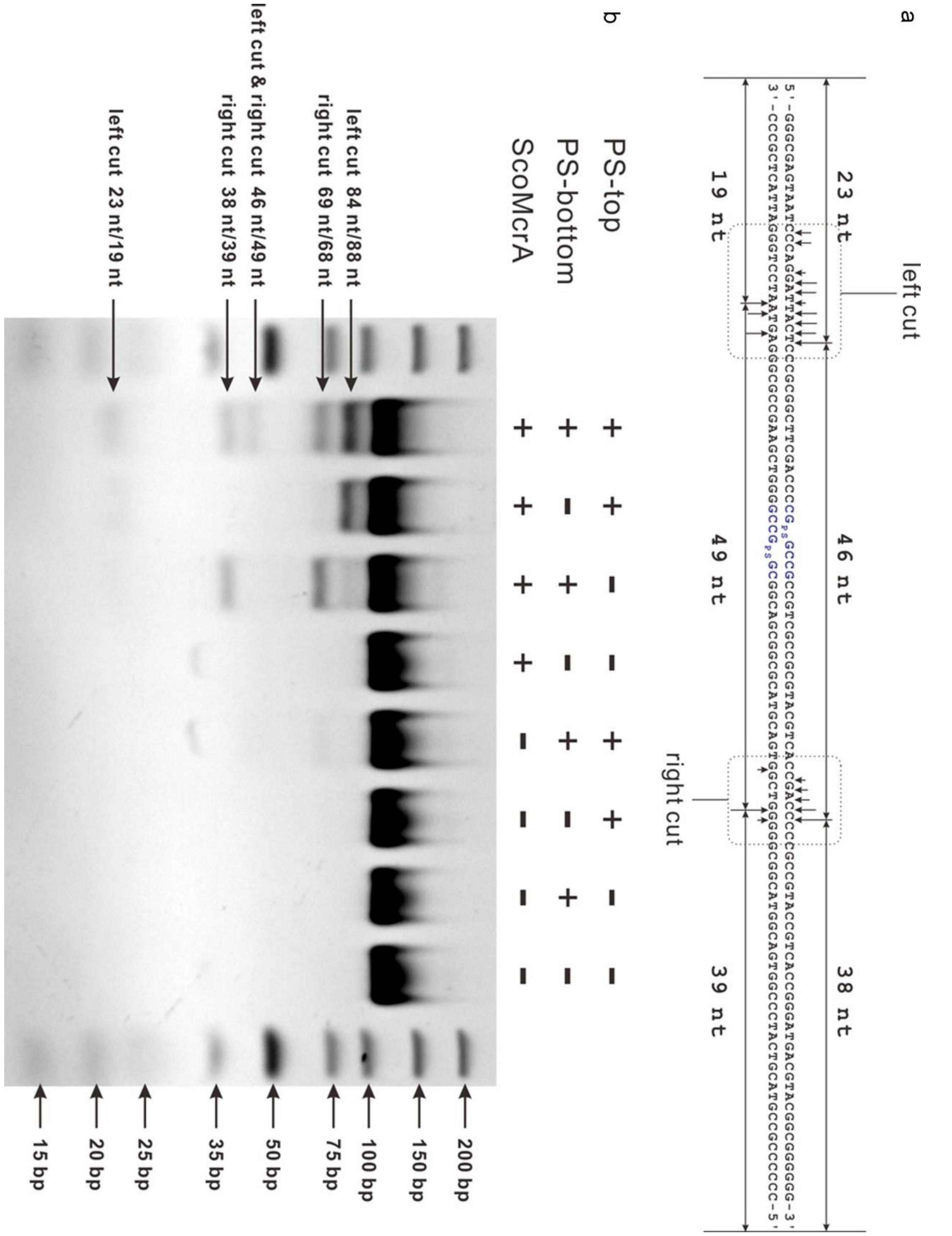

Supplementary Figure 23.

With its SBD domain recognizing the core sequence of PT-DNA, ScoMcrA employs its HNH domain to perform the cleavage ~23 bp away from the phosphorothioation site. (**a**) Sequence of the PT-DNA (with both top and bottom strands phosphorothioated) used in our *in vitro* ScoMcrA cleavage assay. (**b**) *In vitro* ScoMcrA cleavage assay of PT-DNA.

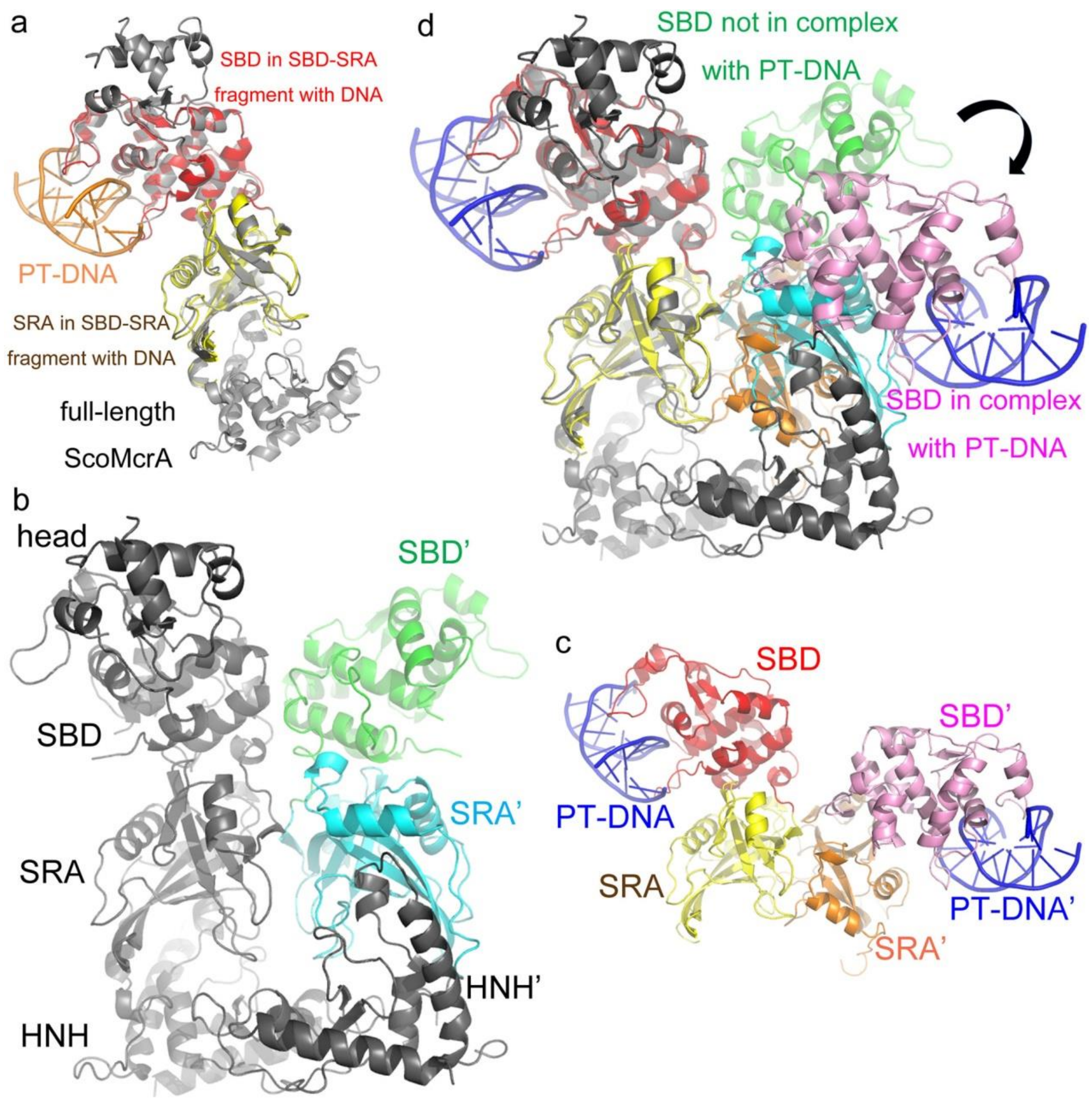

Supplementary Figure 24.

Comparison of full-length ScoMcrA with the SBD-SRA fragment in complex with PT-DNA. (**a**) Comparison of a protomer of full-length ScoMcrA with a protomer of the SBD-SRA fragment in complex with PT-DNA. The SBD and SRA domains in the SBD-SRA fragment are colored in red and yellow, respectively. PT-DNA is colored in orange. Full-length ScoMcrA is colored in grey. (**b**) Structure of the full-length ScoMcrA dimer. The spatial position of one of the protomers is the same as in **a**. The SBD and SRA domains of the other protomer (labeled as SBD′ and SRA′) are colored in green and cyan, respectively. (**c**) Structure of the SBD-SRA fragment in complex with PT-DNA. The spatial position of one of the protomers is the

same as in **a**. (**d**) Superimposition of the full-length ScoMcrA dimer with the SBD-SRA fragment dimer in complex with PT-DNA. Color coding is the same as in **b** and **c**. The ScoMcrA dimer has to undergo a conformational change to adjust its dimeric assembly upon interaction with PT-DNA.

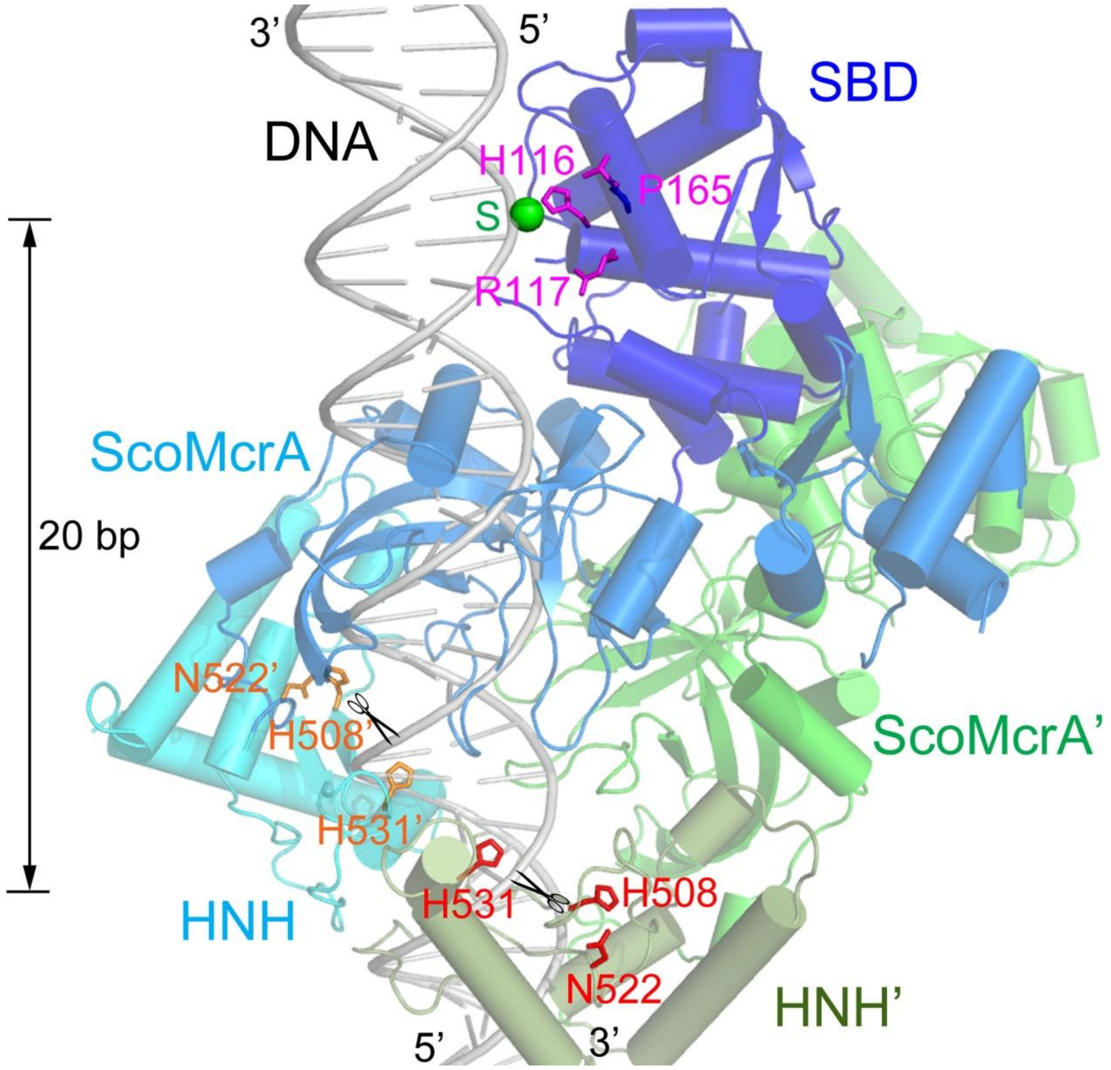

Supplementary Figure 25.

A model of full-length ScoMcrA dimer in complex with PT-DNA. One ScoMcrA protomer is colored in cyan, with its SBD domain colored in blue. The other ScoMcrA protomer (labeled as ScoMcrA′) is colored in green, with its HNH domain (labeled as HNH′) colored in smudge. The H116, R117, and P165 residues of the SBD domain responsible for recognizing the sulfur atom of PT-DNA are shown as magenta sticks. The H508, N522, and H531 residues from the active sites of the HNH and HNH′ domains are shown as orange and red sticks, respectively. The distance between the SBD-recognition site and the HNH′-contact site on PT-DNA is about 20 base pairs. The HNH and HNH′ domains from the two ScoMcrA protomers are responsible for

cleaving the two strands of PT-DNA. The cleavage sites on the two strands of PT-DNA are mimicked according to the most frequently cut sites by the cleavage statistics on the left side of the PT site (see **Supplementary Fig. 23**).

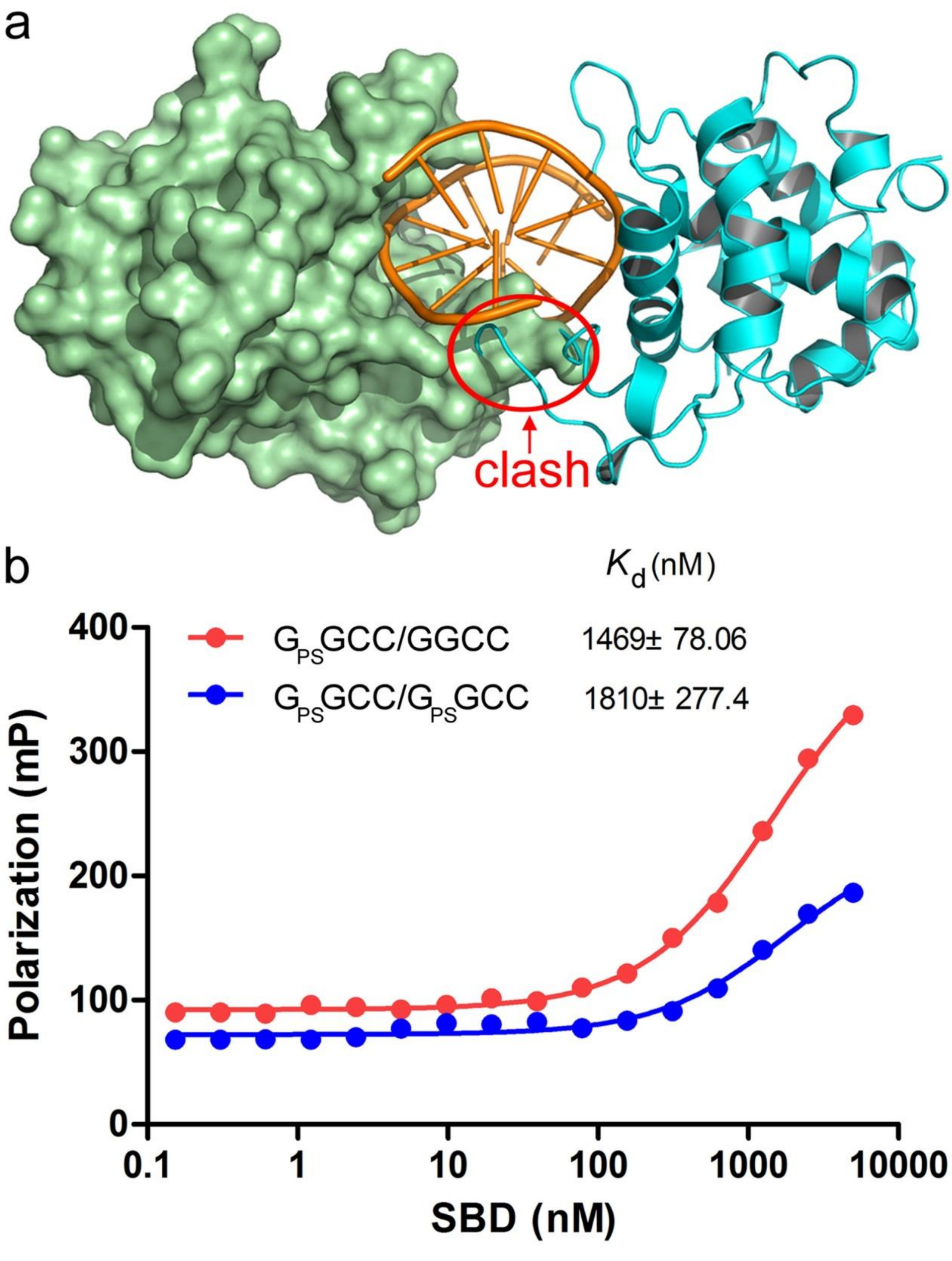

Supplementary Figure 26.

The SRGRR loops from two ScoMcrA–SBD domains cannot bind to the two strands of the same PT-DNA at the same time. (**a**) The SRGRR loops from two ScoMcrA–SBD domains would clash with each other if they are modeled onto the two strands of the same PT-DNA. (**b**) The dissociation constant (K_d) value of ScoMcrA–SBD associating with hemi-phosphorothioated DNA and that of

ScoMcrA–SBD associating with DNA phosphorothioated on both strands are similar. The error values were the standard error of the mean, and were calculated from two to four parallel experiments.

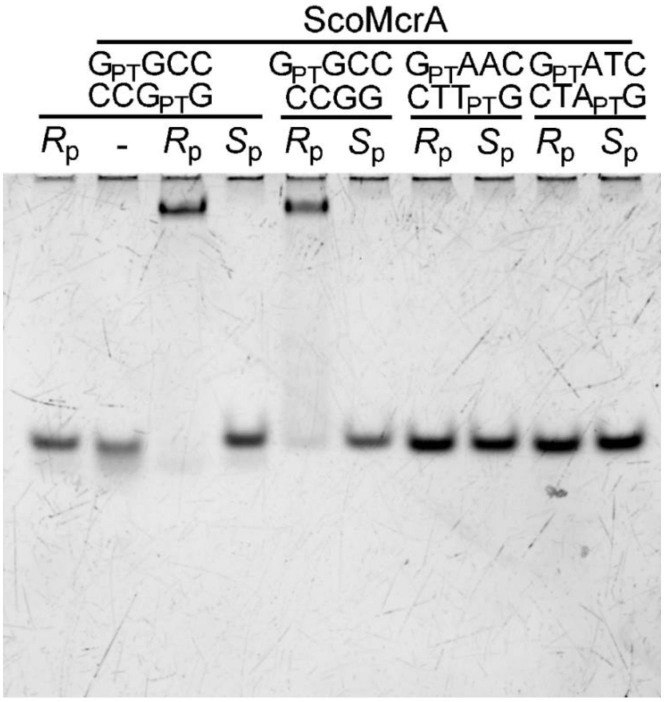

Supplementary Figure 27.

Uncropped image for Figure 1a in the main article.

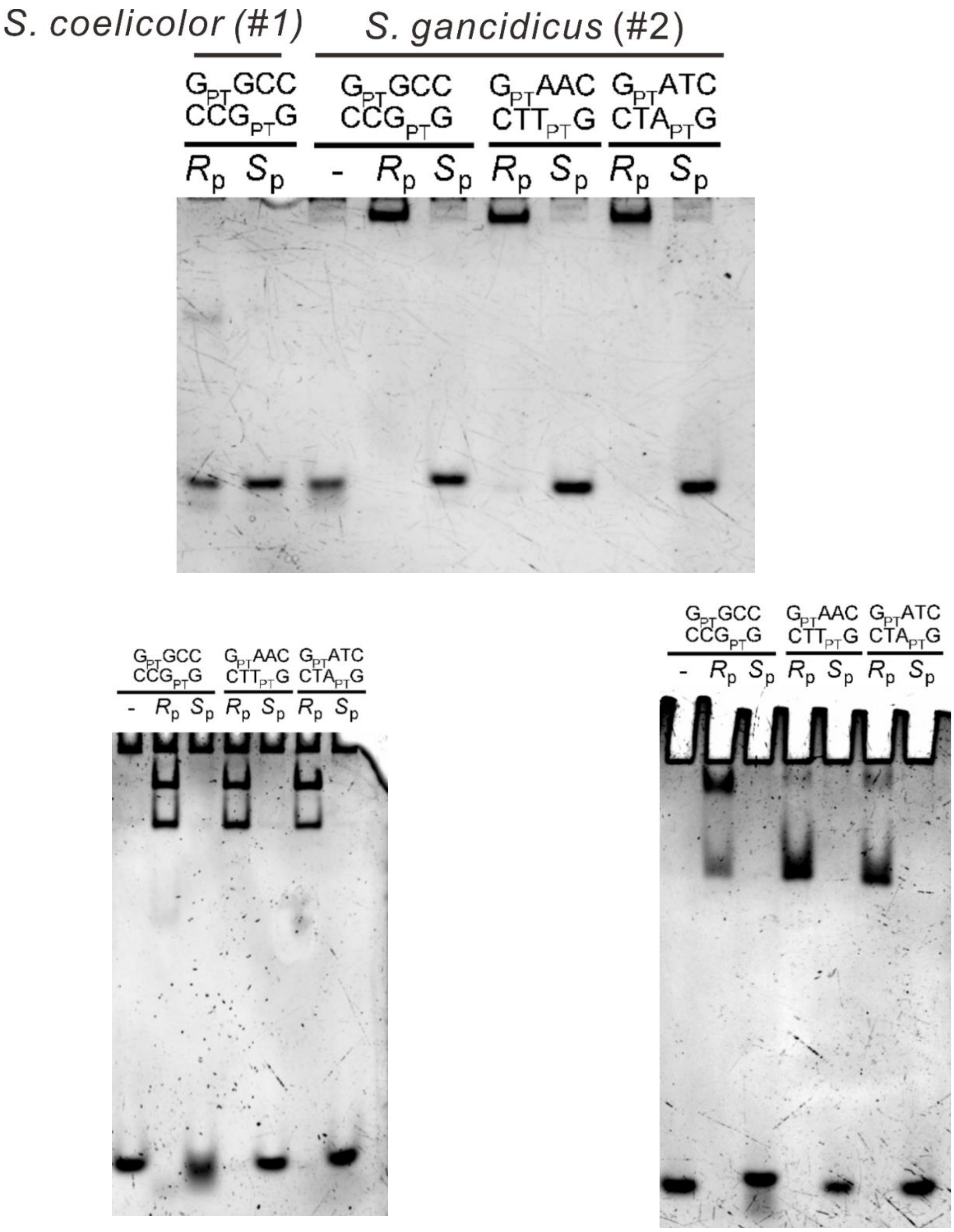

Supplementary Figure 28.

Uncropped images for Figure 6c in the main article.

Supplementary Table 1. Data collection and refinement statistics.

**Data collection**	SeMet-ScoMcrA	Full-length ScoMcrA by itself	The ScoMcrA–SBD-SRA /PT-DNA complex	The ScoMcrA–SBD/PT-DNA complex
Beamline	BL17U1	BL17U1	BL17U1	BL19U1
Space group	$P2_12_12_1$	$P2_12_12_1$	$I4_122$	$C2_1$
Wavelength (Å)	0.97931	0.97892	0.97916	0.97853
Unit cell parameters				
a, *b*, *c* (Å)	130.2, 139.4, 281.0	130.2, 139.4, 281.0	122.3, 122.3, 313.7	88.0, 57.2, 42.3
α, β, γ (°)	90, 90, 90	90, 90, 90	90, 90, 90	90, 101.4, 90
molecules/asymmetric unit	6	6	1	1
Resolution range (Å)	50.0-3.20 (3.31-3.20)	50-3.15 (3.26-3.15)	50-3.30 (3.42-3.30)	50-1.70 (1.76-1.70)
Completeness (%)	99.9 (100.0)	99.9 (99.9)	99.9 (100.0)	97.0 (96.1)
Redundancy	7.3 (7.4)	5.7 (5.7)	3.8 (3.9)	3.8 (3.8)
Total observations	608,722	503,893	132,810	85,089
Unique reflections	83,384	88,897	34,877	22,244
R_{merge} (%)	12.8 (74.0)	14.7 (95.8)	8.5 (63.2)	8.4 (18.8)
I/σ_I	16.8 (3.1)	12.8 (2.1)	14.1 (2.0)	11.4 (8.4)
**Refinement**				
Resolution range (Å)		50-3.15	50-3.30	50-1.70
R_{work}/R_{free} factors (%)		25.4/28.0	23.7/26.3	20.0/21.7
Overall B factor		76.9	101.6	26.2
RMSD bond lengths (Å)		0.011	0.009	0.006
RMSD bond angles (°)		1.480	1.412	1.533
Final model (protein/DNA/solvent atoms)		23,273/0/79	2,527/404/23	1,260/328/172
Ramachandran plot (preferred, generally allowed, disallowed, %)		94.7, 5.0, 0.2	92.2, 6.6, 1.2	98.0, 2.0, 0
PDB code		5ZMM	5ZMN	5ZMO

$R_{merge} = \Sigma_h \Sigma_i |I_{h,i} - I_h| / \Sigma_h \Sigma_i I_{h,i}$ for the intensity (I) of observation i of reflection h. R factor = $\Sigma||F_{obs}| - |F_{calc}|| / \Sigma|F_{obs}|$, where F_{obs} and F_{calc} are the observed and calculated structure factors, respectively. R_{free} = R factor calculated using 5% of the reflection data chosen randomly and omitted from the start of refinement. RMSD, root-mean-square deviation from ideal geometry. Data for the highest resolution shell are shown in parentheses.

Supplementary Table 2. ScoMcrA–SBD homologues are widely present in prokaryotes.

Phylum	Class	Order	Family	Representative organism	Representative protein
Proteobacteria	Alphaproteobacteria	Rhodobacterales	Rhodobacteraceae	*Oceanicola* sp. HL-35	WP_051469220
Proteobacteria	Betaproteobacteria	Burkholderiales	Alcaligenaceae	*Achromobacter* sp. ATCC13047	CUJ22279
Proteobacteria	Betaproteobacteria	Burkholderiales	Burkholderiaceae	*Burkholderia catudaia*	SAK40244
Proteobacteria	Betaproteobacteria	Burkholderiales	Comamonadaceae	*Variovorax* sp. WDL1	KWT70824
Proteobacteria	Betaproteobacteria	Neisseriales	Neisseriaceae	*Kingella kingae*	WP_038329911
Proteobacteria	Betaproteobacteria	Rhodocyclales	Rhodocyclaceae	*Thauera linaloolentis* 47Lol	ENO88419
Proteobacteria	Betaproteobacteria	unclassified	Accumulibacter	*Accumulibacter* sp. SK-11	EXI66531
Proteobacteria	Gammapoteobacteria	Alteromonadales	Alteromonadaceae	*Alteromonas mediterranea*	WP_071958283
Proteobacteria	Gammapoteobacteria	Alteromonadales	Colwelliaceae	*Colwellia* sp. TT2012	WP_057831072
Proteobacteria	Gammapoteobacteria	Alteromonadales	Idiomarinaceae	*Idiomarina woesei*	WP_055439006
Proteobacteria	Gammapoteobacteria	Alteromonadales	Pseudoalteromonadaceae	*Pseudoalteromonas luteoviolacea*	WP_063360449
Proteobacteria	Gammapoteobacteria	Alteromonadales	Psychromonadaceae	*Psychromonas aquimarina*	WP_028862783
Proteobacteria	Gammapoteobacteria	Alteromonadales	Shewanellaceae	*Shewanella violacea*	WP_013051377
Proteobacteria	Gammapoteobacteria	Chromatiales	Chromatiaceae	*Thioflavicoccus mobilis*	WP_041603764
Proteobacteria	Gammapoteobacteria	Chromatiales	Ectothiorhodospiraceae	*Thioalkalivibrio* sp. HK1	WP_025770107
Proteobacteria	Gammapoteobacteria	Cellvibrionales	Halieaceae	*Congregibacter litoralis*	WP_023660089
Proteobacteria	Gammapoteobacteria	Enterobacteriales	Enterobacteriaceae	*Escherichia coli*	WP_000199891
Proteobacteria	Gammapoteobacteria	Methylococcales	Methylococcaceae	*Methylomicrobium buryatense*	WP_017840088
Proteobacteria	Gammapoteobacteria	Methylococcales	Methylothermaceae	*Methylohalobius crimeensis*	WP_022949730
Proteobacteria	Gammapoteobacteria	Oceanospirillales	Alcanivoracaceae	*Alcanivorax* sp. NBRC 102024	WP_062817540
Proteobacteria	Gammapoteobacteria	Oceanospirillales	Hahellaceae	*Endozoicomonas atrinae*	WP_066017028

Proteobacteria	Gammapoteobacteria	Oceanospirillales	Halomonadaceae	*Halomonadaceae* bacterium T82-2	KXS37198
Proteobacteria	Gammapoteobacteria	Oceanospirillales	Oleiphilaceae	*Oleiphilus*	WP_068739908
Proteobacteria	Gammapoteobacteria	Orbales	Orbaceae	*Gilliamella apicola*	WP_065613607
Proteobacteria	Gammapoteobacteria	Thiotrichales	Thiotrichaceae	*Thiothrix lacustris*	WP_028490072
Proteobacteria	Gammapoteobacteria	Vibrionales	Vibrionaceae	*Vibrio parahaemolyticus*	WP_025551667
Proteobacteria	Deltaproteobacteria	Bdellovibrionales	unclassified	*Bdellovibrionales* bacterium GWA2_49_15	OFZ13379
Proteobacteria	Deltaproteobacteria	Desulfobacterales	Desulfobacteraceae	*Desulfotignum phosphitoxidans*	WP_006968875
Proteobacteria	Deltaproteobacteria	Desulfobacterales	Desulfobulbaceae	*Desulfobulbaceae* bacterium BRH_c16a	KJS03538
Proteobacteria	Deltaproteobacteria	Desulfovibrionales	Desulfohalobiaceae	*Desulfonatronovibrio magnus*	WP_052507323
Proteobacteria	Deltaproteobacteria	Desulfovibrionales	Desulfomicrobiaceae	*Desulfomicrobium escambiense*	WP_051306881
Proteobacteria	Deltaproteobacteria	Desulfovibrionales	Desulfonatronaceae	*Desulfonatronum thioautotrophicum*	WP_052813169
Proteobacteria	Deltaproteobacteria	Desulfovibrionales	Desulfovibrionaceae	*Halodesulfovibrio aestuarii*	WP_020001911
Proteobacteria	Deltaproteobacteria	Desulfuromonadales	Desulfuromonadaceae	*Pelobacter carbinolicus*	WP_011339968
Proteobacteria	Deltaproteobacteria	Desulfuromonadales	Geobacteraceae	*Geobacter anodireducens*	WP_066357589
Proteobacteria	Deltaproteobacteria	Myxococcales	Cystobacteraceae	*Cystobacter violaceus*	WP_043403759
Proteobacteria	Deltaproteobacteria	Syntrophobacterales	Syntrophaceae	*Syntrophus* sp. RIFOXYC2_FULL_54_9	OHE31986
Actinobacteria	Rubrobacteria	Rubrobacterales	Rubrobacteraceae	*Rubrobacter radiotolerans*	AHY45647
Actinobacteria	Actinobacteria	Streptomycetales	Streptomycetaceae	*Streptomyces pristinaespiralis*	WP_053557345
Actinobacteria	Actinobacteria	Geodermatophilales	Geodermatophilaceae	*Blastococcus saxobsidens*	WP_014377222
Actinobacteria	Actinobacteria	Frankiales	Frankiaceae	*Frankia inefficax*	WP_013424985
Actinobacteria	Actinobacteria	Kineosporiales	Kineosporiaceae	*Kineococcus radiotolerans*	WP_012084822
Actinobacteria	Actinobacteria	Micrococcales	Microbacteriaceae	*Microbacterium* sp. SUBG005	KEP73612

Actinobacteria	Actinobacteria	Micrococcales	Micrococcaceae	*Arthrobacter* sp. Leaf137	WP_056082307
Actinobacteria	Actinobacteria	Propionibacteriales	Nocardioidaceae	*Actinopolymorpha singaporensis*	SDR88613
Actinobacteria	Actinobacteria	Streptosporangiales	Streptosporangiaceae	*Herbidospora cretacea*	WP_051759891
Actinobacteria	Actinobacteria	Streptosporangiales	Nocardiopsaceae	*Nocardiopsis valliformis*	WP_017578716
Actinobacteria	Actinobacteria	Streptosporangiales	Thermomonosporaceae	*Actinomadura rubrobrunea*	WP_067908863
Actinobacteria	Actinobacteria	Micromonosporales	Micromonosporaceae	*Micromonospora rhizosphaerae*	SCL20333
Actinobacteria	Actinobacteria	Pseudonocardiales	Pseudonocardiaceae	*Saccharothrix espanaensis*	WP_051075824
Actinobacteria	Actinobacteria	Corynebacteriales	Nocardiaceae	*Nocardia tenerifensis*	WP_051187150
Actinobacteria	Actinobacteria	Corynebacteriales	Mycobacteriaceae	*Mycobacterium smegmatis*	WP_014877012
Bacteroidetes	Bacteroidia	Bacteroidales	Bacteroidaceae	*Bacteroides salyersiae*	WP_005930485
Bacteroidetes	Bacteroidia	Bacteroidales	Marinilabiaceae	*Marinilabilia salmonicolor*	WP_010662388
Bacteroidetes	Bacteroidia	Bacteroidales	Porphyromonadaceae	*Dysgonomonas* sp. BGC7	WP_050699841
Bacteroidetes	Bacteroidia	Bacteroidales	Prolixibacteraceae	*Draconibacterium orientale*	WP_051567711
Bacteroidetes	Bacteroidia	Bacteroidales	Prevotellaceae	*Prevotella* sp. P6B4	WP_028903769
Bacteroidetes	Flavobacteriia	Flavobacteriales	Crocinitomicaceae	*Fluviicola* sp. RIFCSPHIGHO2_02_FULL_43_260	OGS76951
Bacteroidetes	Flavobacteriia	Flavobacteriales	Flavobacteriaceae	*Flavobacterium filum*	WP_051220749
Bacteroidetes	Sphingobacteria	Sphingobacteriales	Sphingobacteriaceae	*Pedobacter* sp. CCM 8644	WP_068823013
Bacteroidetes	Sphingobacteria	Sphingobacteriales	Saprospiraceae	*Saprospira grandis*	WP_002658428
Bacteroidetes	Cytophagia	Cytophagales	Hymenobacteraceae	*Hymenobacter* sp. MIMtkLc17	WP_052732857
Bacteroidetes	Cytophagia	Cytophagales	Cyclobacteriaceae	*Algoriphagus machipongonensis*	WP_008200678
Bacteroidetes	Cytophagia	Cytophagales	Cytophagaceae	*Rufibacter tibetensis*	WP_062543367
Bacteroidetes	Cytophagia	Cytophagales	Flammeovirgaceae	*Roseivirga spongicola*	WP_068216431
Bacteroidetes	Rhodothermia	Rhodothermales	Rhodothermaceae	*Salinibacter ruber*	WP_011403317
Bacteroidetes	Balneolia	Balneolales	Balneolaceae	*Balneola* sp. EhC07	WP_066221814

Cyanobacteria	Oscillatoriophycideae	Oscillatoriales	Gomontiellaceae	*Crinalium epipsammum*	WP_015202527
Cyanobacteria	Oscillatoriophycideae	Oscillatoriales	Microcoleaceae	*Planktothrix rubescens*	WP_026788000
Cyanobacteria	Oscillatoriophycideae	Oscillatoriales	Oscillatoriaceae	*Lyngbya confervoides*	WP_052288534
Cyanobacteria	Oscillatoriophycideae	Chroococcales	Aphanothecaceae	*Crocosphaera watsonii*	WP_007312075
Cyanobacteria	Oscillatoriophycideae	Chroococcales	Microcystaceae	*Microcystis aeruginosa*	WP_066030138
Cyanobacteria		Nostocales	Aphanizomenonaceae	*Aphanizomenon flos-aquae*	WP_027404587
Cyanobacteria		Nostocales	Hapalosiphonaceae	*Fischerella* sp. PCC 9605	WP_035140596
Cyanobacteria		Nostocales	Nostocaceae	*Nostoc* sp. KVJ20	WP_069068098
Cyanobacteria		Nostocales	Symphyonemataceae	*Mastigocladopsis repens*	WP_017315877
Cyanobacteria		Nostocales	Tolypothrichaceae	*Tolypothrix bouteillei*	WP_038072579
Cyanobacteria		Synechococcales	Leptolyngbyaceae	*Leptolyngbya* sp. Heron Island J	WP_023071809
Cyanobacteria		Synechococcales	Pseudanabaenaceae	*Pseudanabaena* sp. PCC 6802	WP_019500934
Cyanobacteria		Synechococcales	Synechococcaceae	*Synechococcus* sp. PCC 8807	WP_065717476
Firmicutes	Clostridia	Clostridiales	Clostridaceae	*Alkaliphilus transvaalensis*	WP_026477587
Firmicutes	Clostridia	Clostridiales	Peptostreptococcaceae	*Desulfotomaculum kuznetsovii*	WP_013821698
Firmicutes	Clostridia	Clostridiales	Peptococcaceae	*Fusibacter* sp. 3D3	WP_069875450
Firmicutes	Clostridia	Clostridiales	Syntrophomonadaceae	*Syntrophomonas palmitatica*	WP_054693267
Firmicutes	Clostridia	Thermoanaerobacterales	Thermoanaerobacteriaceae	*Thermacetogenium phaeum*	WP_015050782
Firmicutes	Bacilli	Bacillales	Bacillaceae	*Bacillus cohnii*	WP_066411193
Firmicutes	Bacilli	Bacillales	Paenibacillaceae	*Paenibacillus* sp. DMB20	WP_046678394
Firmicutes	Bacilli	Bacillales	Planococcaceae	[*Bacillus*] *aminovorans*	WP_063974911
Chloroflexi	Chloroflexia	Herpetosiphonales	Herpetosiphonaceae	*Herpetosiphon geysericola*	WP_054535302
Chloroflexi	Chloroflexia	Chloroflexales	Oscillochloridaceae	*Candidatus Chloroploca asiatica*	WP_097655259
Chloroflexi	Anaerolineae	Anaerolineales	Anaerolineaceae	*Anaerolinea thermolimosa*	WP_062196486
Planctomycetes	Planctomycetacia	Brocadiales	Brocadiaceae	Candidatus *Brocadia sinica*	WP_052562946

Planctomycetes	Planctomycetacia	Planctomycetales	Planctomycetaceae	*Rhodopirellula maiorica*	WP_040764095
Planctomycetes	Planctomycetacia	Planctomycetales	Isosphaeraceae	*Singulisphaera sp. GP187*	WP_083670156
Planctomycetes	Planctomycetacia	Planctomycetales	Gemmataceae	*Gemmata sp. SH-PL17*	WP_082838880
Planctomycetes	Planctomycetia	unclassified	unclassified	*Planctomycetia bacterium 21-64-5*	OYV96022
Verrucomicrobia	Verrucomicrobiae	Verrucomicrobiales	Verrucomicrobiaceae	*Verrucomicrobium* sp. BvORR034	WP_050026552
Verrucomicrobia	Verrucomicrobiae	Verrucomicrobiales	Rubritaleaceae	*Rubritalea marina*	WP_018969043
Verrucomicrobia	Opitutae	unclassified	unclassified	*Opitutae bacterium Tous-C1TDCM*	PAW63037
Acidobacteria	Holophagae	Holophagales	Holophagaceae	*Holophaga foetida*	WP_026939014
Nitrospirae	unclassified	unclassified	unclassified	Nitrospirae bacterium GWC2_57_9	OGW47409
Deinococcus-Thermus	Deinococci	Thermales	Thermaceae	*Meiothermus timidus*	WP_084784957
	Deinococci	Deinococcales	Deinococcaceae	*Deinococcus sp. NW-56*	WP_104989995
Balneolaeota	Balneolia	Balneolales	Balneolaceae	*Balneola sp. EhC07*	WP_066221814
Rhodothermaeota	Rhodothermia	Rhodothermales	Rubricoccaceae	*Rubrivirga marina*	WP_095509486
Lentisphaerae	unclassified	unclassified	unclassified	Lentisphaerae bacterium RIFOXYC12_FULL_60_16	OGV61158

ScoMcrA–SBD homologues were found in at least fourteen bacterial phyla: proteobacteria, actinobacteria, bacteroidetes, firmicutes, cyanobacteria, chloroflexi, planctomycetes, verrucomicrobia, acidobacteria, nitrospirae, deinococcus-thermus, rhodothermaeota, balneolaeota, and lentisphaerae. The names of phyla, classes, orders, and families in which proteins containing ScoMcrA–SBD-like domains were found by BLAST search are listed, as well as the names of representative organisms and the accession numbers of representative ScoMcrA–SBD-like proteins.

Supplementary Table 3. Representative ScoMcrA–SBD homologues used for multiple sequence alignment in Supplementary Fig. 15.

	Phylum	Class	Order	Family	Organism	Protein
1	Actinobacteria	Actinobacteria	Streptomycetales	Streptomycetaceae	*Streptomyces coelicolor*	ScoMcrA
2	Actinobacteria	Actinobacteria	Streptomycetales	Streptomycetaceae	*Streptomyces gancidicus*	WP_006134840
3	Actinobacteria	Actinobacteria	Streptomycetales	Streptomycetaceae	*Streptomyces hygroscopicus*	WP_014674231
4	Actinobacteria	Actinobacteria	Streptosporangiales	Nocardiopsaceae	*Nocardiopsis dassonvillei*	WP_013156064
5	Proteobacteria	Gammaproteobacteria	Enterobacterales	Enterobacteriaceae	*Escherichia coli*	WP_000199891
6	Proteobacteria	Gammaproteobacteria	Enterobacterales	Morganellaceae	*Morganella morganii*	WP_032098169
7	Proteobacteria	Gammaproteobacteria	Enterobacterales	Enterobacteriaceae	*Salmonella enterica*	WP_061382821
8	Proteobacteria	Gammaproteobacteria	Vibrionales	Vibrionaceae	*Vibrio cholerae*	WP_001896471
9	Proteobacteria	Gammaproteobacteria	Alteromonadales	Shewanellaceae	*Shewanella violacea*	WP_013051377
10	Proteobacteria	Betaproteobacteria	Neisseriales	Neisseriaceae	*Eikenella corrodens*	WP_003822245
11	Proteobacteria	Deltaproteobacteria	Desulfuromonadales	Geobacteraceae	*Geobacter sulfurreducens*	WP_045668470
12	Firmicutes	Bacilli	Bacillales	Bacillaceae	*Bacillus megaterium*	WP_013083516
13	Firmicutes	Clostridia	Clostridiales	Clostridaceae	*Alkaliphilus metalliredigens*	WP_012063279
14	Cyanobacteria		Nostocales	Aphanizomenonaceae	*Nodularia spumigena*	WP_006195419
15	Cyanobacteria	Oscillatoriophycideae	Oscillatoriales	Microcoleaceae	*Arthrospira maxima*	WP_006670171
16	Cyanobacteria		Synechococcales	Synechococcaceae	*Synechococcus* sp. PCC 7117	WP_065711998
17	Chloroflexi	Chloroflexia	Herpetosiphonales	Herpetosiphonaceae	*Herpetosiphon aurantiacus* DSM 785	ABX03229
18	Bacteroidetes	Bacteroidia	Bacteroidales	Bacteroidaceae	*Bacteroides fragilis*	WP_005785611
19	Bacteroidetes	Bacteroidia	Bacteroidales	Prevotellaceae	*Prevotella ruminicola*	WP_041385766
20	Bacteroidetes	Cytophagia	Cytophagales	Cytophagaceae	*Flexibacter litoralis*	WP_014797641
21	Planctomycetes	Planctomycetacia	Candidatus Brocadiales	Candidatus Brocadiaceae	*Candidatus Kuenenia stuttgartiensis*	CAJ72745
22	Verrucomicrobia	Verrucomicrobiae	Verrucomicrobiales	Verrucomicrobiaceae	*Verrucomicrobium* sp. BvORR034	WP_050026552

23	Acidobacteria	Holophagae	Holophagales	Holophagaceae	*Holophaga foetida*	WP_026939014

The names of the organisms they originate from, as well as the phylum, class, order, and family which each organism belongs to, are listed.

Supplementary Table 4. Strains, plasmids, primers and oligonucleotides used in this study.

Name	Description	Source or references
Escherichia coli		
DH10B	F^- *mcr*A Δ(*mrr*- *hsd*RMS- *mcr*BC) φ80*lac*ZΔM15 Δ*lac*X74 *rec*A1 *end*A1 *ara*D139 Δ(*ara*, *leu*)7697 *gal*U *gal*K λ^- *rps*L *nup*G	Thermo Fisher
BL21(DE3)	F^- *ompT hsdS*$_b$ (r_b^- *(2)*m_b^-) *gal dcm* (DE3) pLysE	Novagen
Plasmid		
pSJ8	pET43.1a derived protein expression vector, MBP tag, TEV protease cleavage site (ENLYFQG), Ampicillin resistance	Dr. Jiahai Zhou, unpublished data
pJTU1660	pSJ8 derivative for heterologous expression and purification of ScoMcrA	This study
pJTU1668	pSJ8 derivative for heterologous expression and purification of SBD-SRA	This study
pJTU1669	pSJ8 derivative for heterologous expression and purification of SBD	This study
pJTU1670	pSJ8 derivative for heterologous expression and purification of SBD domain of *E. coli* homolog	This study
pJTU1671	pSJ8 derivative for heterologous expression and purification of SBD domain of *M. morganii* homolog	This study
pJTU1672	pSJ8 derivative for heterologous expression and purification of full-length *S. gancidicus* homolog	This study
pSBD-H116I	a mutant of pJTU1669 with the product of H116I	This study
pSBD-R117A	a mutant of pJTU1669 with the product of R117A	This study
pSBD-R117G	a mutant of pJTU1669 with the product of R117G	This study
pSBD-Y164I	a mutant of pJTU1669 with the product of Y164I	This study
pSBD-P165N	a mutant of pJTU1669 with the product of P165N	This study
pSBD-A168G	a mutant of pJTU1669 with the product of A168G	This study
pSBD-A168I	a mutant of pJTU1669 with the product of A168I	This study
pSBD-Y164F	a mutant of pJTU1669 with the product of Y164F	This study

pSBD-S187A	a mutant of pJTU1669 with the product of S187A	This study
pSBD-R191A	a mutant of pJTU1669 with the product of R191A	This study
pBluescript II SK$^+$	Standard cloning vector, Ampicillin resistance, *col*E1 origin	Stratagene
pJTU1238	pBluescript II SK$^+$ derivative carrying the *dnd* gene cluster from *Salmonella enterica* serovar Cerro 87 which makes PT modification in the pattern of G$_{PS}$AAC/G$_{PS}$TTC	Xu et al., 2010
pACYCDuet™-1	coexpression vector, compatible with pBluescript II SK+, chloramphenicol resistance, P15A origin	Novagen
pJTU1673	pACYCDuet™-1 derivative carrying full-length *E. coli* homolog with promotor	This study
pJTU1674	pACYCDuet™-1 derivative carrying full-length *M. morganii* homolog with *E. coli* promotor	This study
pJTU1675	pACYCDuet™-1 derivative carrying full-length *S. gancidicus* homolog with *E. coli* promotor	This study
**Primers**		
ScoFL-F	AAAGATCTGCACCTTCGGAGATCACTCGT	
ScoFL-R	TTGAATTCTCACTGGAGTTTCTCTTCGTG	
SBD-F	AAGGATCCAGGGAGGCCCCCAA	
SBD-R	AAGAATTCTCACGGCCGCAGAGCAT	
SBDSRA-F	AAAGATCTAGGGAGGCCCCCAA	
SBDSRA-R	AAGAATTCTCATCGCCTGTAAGCTT	
Eco-SBD-F	AAGGATCCACCTCTGACAAAACCCT	
Eco-SBD-R	TTGAATTCTCAGCGAAACTTCGGATCGCG	
Mmo-SBD-F	AAGGATCCATTAGCCCGGAAAC	
Mmo-SBD-R	TTGAATTCTCAACGGAAATGCGGATCGCG	
Sga-F	AAGGATCCGGCGCAGAAGCTGG	
Sga-R	TTGAATTCTTAGCTGGCCGCCGGT	
H116I-F	GTCCAGCCCTCATCCGCCCCGTTCT	
H116I-R	AGAACGGGGCGGATGAGGGCTGGAC	
R117A-F	CAGCCCTCCACGCCCCCGTTCTTCT	

R117A-R	AGAAGAACGGGGGCGTGGAGGGCTG
R117G-F	CAGCCCTCCACGGCCCCGTTCTTCT
R117G-R	AGAAGAACGGGGCCGTGGAGGGCTG
Y164I-F	ATGGGGTGCGCATCCCATTCTGGGC
Y164I-R	GCCCAGAATGGGATGCGCACCCCAT
P165N-F	GGGTGCGCTACAATTTCTGGGCCCT
P165N-R	AGGGCCCAGAAATTGTAGCGCACCC
A168G-F	ACCCATTCTGGGGCCTCGTGCGCGA
A168G-R	TCGCGCACGAGGCCCCAGAATGGGT
A168I-F	ACCCATTCTGGATCCTCGTGCGCGA
A168I-R	TCGCGCACGAGGATCCAGAATGGGT
Y164F-F	ATGGGGTGCGCTTCCCATTCTGGGC
Y164F-R	GCCCAGAATGGGAAGCGCACCCCAT
S187A-F	CACTCTGACTGCTCGAGGGCGTCGC
S187A-R	GCGACGCCCTCGAGCAGTCAGAGTG
R191A-F	AGTCGAGGGCGTGCCCCCACCCTGG
R191A-R	CCAGGGTGGGGGCACGCCCTCGACT
EcoHE-F	GCTACGGGTGATGGGTTATGAC
EcoHE-R	TTGAATTCTCAATGCTCTCCTCTG
P-Mmo-R	GCAGGGTTTCCGGGCTAATCATTGCGTTGGAGAATCCATGGGCACA
MmoHE-F	ATGATTAGCCCGGAAACCCTGC
MmoHE-R	TTGAATTCTCAGACTTTGAAAACT
P-Sga-R	TTTCACCAGCTTCTGCGCCCATTGCGTTGGAGAATCCATGGGCACA
SgaHE-F	ATGGGCGCAGAAGCTGGTGAAA
SgaHE-R	TTGAATTCTTAGCTGGCCGCCGGT

Oligonucleotides

GGCC10	CCCG$_{PS}$GCCGGG
GGCC8	CCG$_{PS}$GCCGG
GAAC-S	GGCG$_{PS}$AACGTG
GAAC-A	CACG$_{PS}$TTCGCC
GATC-S	GATG$_{PS}$ATCCTA
GATC-A	TAGG$_{PS}$ATCATC
S10hemi-S	CCCG$_{PS}$GCCGCC
U10hemi-S	CCCGGCCGCC
U10hemi-A	GGCGGCCGGG
CUThemi-s1	GGGCGAGTAATCCCAGGATTACTCCCGCGGCTTCGAC
CUThemi-s2	CCCG$_{PS}$GCCGCCGT
CUThemi-s3	CGCCGCGTACGTCACCGACCCCCGCCGTACGTCACCGGGATGACGTACGGCGGGGGG
CUThemi-a1	GGGTCGAAGCCGCGGGAGTAATCCTGGGATTACTCGCCC
CUThemi-a2	GGCG$_{PS}$GCCG
CUThemi-a3	CCCCCCGCCGTACGTCATCCCGGTGACGTACGGCGGGGGTCGGTGACGTACGCGGCGAC
CUThemi-s2U	CCCGGCCGCCGT
CUThemi-a2U	GGCGGCCG

sSupplementary Table 5. Codon optimized DNA sequences.

Codon optimized DNA sequence of *M. morganii* ScoMcrA homolog (WP_032098169)
ATGATTAGCCCGGAAACCCTGCAGTCTGCGATTGCCAGCATCTCTGTGTGGCGCCAAGGCGATGTTTGCGCGCCGCATAAACCGCTGCTGCTGCTGTATGTTC<br>TGTCACAGTACAAAGCCGGCCATCCGCGCCTGTTTAATTATGGTCGTGAAATCCACGAACCGCTGACCCGTCTGCTGCGCGAATTTGGTCCGAAACGTCGCA<br>CGGATTACCCGAACATGCCGTTTTGGCGTCTGCGCAGTGATTCCTTCTGGGAAATTACGGACGCGGAAGGCTGTAAACATCGTCGCGGTAACACCCAGCCGA<br>CGAAAAAAGAACTGATCGATAATCACGTCTCAGGCGGTTTTGACGAAGCGGCCTATCGTCAGCTGCGCGAACATCCGGAACTGATTGATAAACTGGCACAG<br>CAAATCCTGACCAACCGCTTCCCGGAATCGATTCAGCGTATCCTGGCTAATCAACTGGGCCTGGATTTTGTCGACCGTACGAAAAACCGCGATCCGCATTTCC<br>GTGACATTATCCTGCGCGCGTATCACAGCCGTTGCGCCTTTTGTGGTTACGATCTGCGTCTGGACGGTGCCCTGGTGGGTATTGAAGCAGCTCATATCCACTG<br>GAAAGCGTATGGCGGCCCGTGCGTGGTTCACAATGGCCTGGCTCTGTGTAGTCTGCATCACGATGCATTTGACATGGGCGCTTTCGGTCTGGATAAAAACCT<br>GACCATTCGTATCTCCGCCGGCGTGTCACGTTCGCCGGTCGTGGATAACCTGTTTTGGCAGCGCAATGGTCAACTGCTGCATCTGCCGCACGACGAATCTCTG<br>TGGCCGGCAGAACAGTACATTGGTTGGCATCGTAAACAAGTTTTCAAAGTCTGA
Codon optimized DNA sequence of *M. morganii* ScoMcrA homolog (WP_032098169)
ATGGGCGCAGAAGCTGGTGAAACCTGCACCACGGCGGCCCTGTGTATGTATGATCTGAGCTACCGTCCGACGTCTCTGCCGCGTATGGGTCTGGGTGACATT<br>CGTCATGAACACGTGATCGCAGCTACCGAAGAATTTCGTCGCCTGGGTCGTGAAGAATTTCTGCGCACGTATGGCTTCGGTCGTGCGCGCTCATACCAGCTG<br>GTTCTGGATGGCCGTCGCTATGACTCGAAAGCGATTGCCGGCGTCGCACATGGTTACGCTACCGGCGATTTTCTGAGTGCAGCAGACTTCTCCGGCGGTGCA<br>GCTACCGTCGCACGTTGCCTGCGCGAACTGGGTTTCGTGGTTGAAACGGGTGCACCGAGTGATAACCGTGCAGGTCTGCTGGCACGTCTGCGTCAGCTGCG<br>TGTTAGCCGTGGTCCGGGTAATCCGGCACCGAGCCGTCATCAACCGCTGTCTCTGCTGTGGGCAATGGCTCGTGCAGCAGATGACCGTCCGCGTCTGACCCC<br>GTGGCCGACGTTTCGCGATGAAGTTGGTCCGCTGCTGGTTGAATTTGGCCTGCCGAACGCGAAAGCCACCCCGGAATATCCGTTTTGGCACCTGCAGGGTTC<br>TGAACTGTGGGAAGTGCGTGGCATCCCGGCAGAACTGGCTATGCGCATGCCGAACGTTGGTCTGTTTAATGAACATCACCCGCAAGCAGGTTTCACCGCAG<br>AAACGGCAGATCTGCTGTGCGACCCGGTGACCCGTCTGGAAGCAATTGCTACCATCTGTGAAACGTATCTGGGCGATATTGACCGTCAGGCGCTGTTCTCAC<br>GCATCGGCCTGTCGGGTTACACCACGGCCGGCGGTCCGCTGACCCCGTGGGCAGAAGAACCGGGCGGTGCTGATACGGACGAATATGGTCGCAGCGCAGG<br>TCCGGCTCCGCGTCGCGAAGCCACCCGTTCTGTCATTGTGCGCGATGAAGCGCTGGCCCGTAAAGTCAAAGAACTGGAAGATGACCGCTGCCAAGTGTGTG<br>ATACCGCACTGCGTTATCTGAATCGCCCGTACAGTGAAGCAGCTCATATCCGCGGCCTGGGTTCCCCGCATCACGGTCCGGATGAACTGCAAAACCTGCTGT<br>GCCTGTGTGCGAATTGCCACGTGCTGTTTGATGGTCTGGAAATTTATGTGGACACCGAAGGTCTGGTTCGTGGTACGCGTGGTGATCGTGCACCGCGTCCGC<br>TGCGTCGCGCACCGGGTCATCCGATCGATGAAGCGCATGTTGCCTACCACCGTGACCTGTGTAACCTGAATGCCCGCAAACCGGCGGCCAGCTAA

Supplementary References

1. Shen, B. W., Landthaler, M., Shub, D. A. & Stoddard, B. L. DNA binding and cleavage by the HNH homing endonuclease I-HmuI. *J. Mol. Biol.* **342**, 43-56 (2004).
2. Shen, B. W., *et al.* Unusual target site disruption by the rare-cutting HNH restriction endonuclease PacI. *Structure* **18**, 734-43 (2010).
3. Xu, S. Y. & Gupta, Y. K. Natural zinc ribbon HNH endonucleases and engineered zinc finger nicking endonuclease. *Nucleic Acids Res.* **41**, 378-90 (2013).
